# Formation of ibrutinib solvates: so similar, yet so different

**DOI:** 10.1107/S2052252523001197

**Published:** 2023-02-24

**Authors:** Jan Jirát, Jan Rohlíček, Jakub Kaminský, Tomáš Jirkal, Luděk Ridvan, Eliška Skořepová, Vít Zvoníček, Michal Dušek, Miroslav Šoóš

**Affiliations:** a Chemical Engineering, University of Chemistry and Technology in Prague, Technická 3, Praha, Czech Republic; b Zentiva, k.s., U kabelovny 130, Prague 10 10237, Czech Republic; c Institute of Physics of the Czech Academy of Sciences, Na Slovance 2, Prague 8 182 00, Czech Republic; d Institute of Organic Chemistry and Biochemistry of the CAS, Flemingovo náměstí 542/2, Prague 6, Czech Republic; University of Iowa, USA

**Keywords:** ibrutinib, multicomponent crystal solvates, transformation mechanisms

## Abstract

The transformation processes of non-solvated ibrutinib into a series of halogenated benzene solvates are explored in detail here. The transformation was studied in real time by X-ray powder diffraction in a glass capillary. Crystal structures of chloro­benzene, bromo­benzene and iodo­benzene solvates are isostructural, whereas the structure of fluoro­benzene solvate is different. Four different mechanisms for transformation were discovered despite the similarity in the chemical nature of the solvents and crystal structures of the solvates formed. These mechanisms include direct transformations and transformations with either a crystalline or an amorphous intermediate phase. The binding preference of each solvate in the crystal structure of the solvates was examined in competitive slurry experiments and further confirmed by interaction strength calculations. Overall, the presented system and online X-ray powder diffraction measurement provide unique insights into the formation of solvates.

## Abstract 2

Four different mechanisms of solvate formation were observed and explained for four solvates of the pharmaceutical substance ibrutinib with the series of solvents fluoro­benzene, chloro­benzene, bromo­benzene and iodo­benzene.

## 1. Introduction

Multicomponent crystals, *i.e.* salts, cocrystals and solvates, have been actively researched for several decades in academia as well as in industry. Academic researchers discovered new multicomponent crystals and have studied the intermolecular interactions within the crystal structures. The arrangement of molecules in crystal structures determines the crystal properties (Aguiar & Zelmer, 1969 ▸; Matsuda *et al.*, 2011 ▸; Zvoníček *et al.*, 2018 ▸; Sun & Grant, 2001 ▸; Kokubo *et al.*, 1987 ▸; Pandit *et al.*, 1984 ▸). Therefore, approaches that can shed new light on molecular interactions within these crystals and their properties have received a lot of attention in recent years. These include both computational and experimental efforts, such as structure–property relations (Suresh *et al.*, 2015 ▸; Stanton & Bak, 2008 ▸; de Moraes *et al.*, 2017 ▸; Arlin *et al.*, 2011 ▸; Collier *et al.*, 2006 ▸), crystal structure prediction (Price, 2014 ▸; Reilly *et al.*, 2016 ▸), intermolecular energy calculations and other types of calculations (Dash & Thakur, 2021 ▸; Musumeci *et al.*, 2011 ▸; Issa *et al.*, 2009 ▸; Karamertzanis *et al.*, 2009 ▸), or systematic screening and rational design of multicomponent crystals with desired properties (Aakeröy & Salmon, 2005 ▸; Sládková *et al.*, 2015 ▸; Desiraju, 1995 ▸; Amrutha *et al.*, 2020 ▸). Various industrial fields also contribute to the fast development of multicomponent crystals. Improvement of physical properties of high-value chemical products has been demonstrated for agrochemicals (Nauha & Nissinen, 2011 ▸), solid explosives (Bolton *et al.*, 2012 ▸) and in particular for pharmaceuticals (Zvoníček *et al.*, 2017 ▸; Billot *et al.*, 2013 ▸; Stanton & Bak, 2008 ▸; Schultheiss & Newman, 2009 ▸).

Tens of thousands of new multicomponent crystals were discovered since the first reported organic:organic cocrystal (quinhydrone) back in 1884 by Wöhler (1844 ▸). The majority of their crystal structures were solved over time [the crystal structure of quinhydrone was solved in 1958, long after its discovery (Matsuda *et al.*, 1958 ▸)]. Chemical and physical properties of new multicomponent crystals are routinely characterized during the screening process in both academia and industry, thus quickly epanding the knowledge and applicability of multicomponent solids.

**Figure 1:**
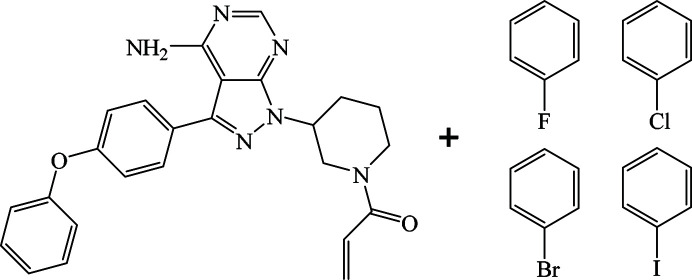
Molecules of ibrutinib and the solvents used (FBZ, CBZ, BBZ and IBZ).

Despite fast progress in the crystal engineering field and the ever-growing datasets, the process of multicomponent crystal formation itself remains inadequately understood. Commonly used methods for characterization of crystalline materials, such as powder X-ray diffraction (PXRD), single-crystal X-ray diffraction (SCXRD), infrared or Raman spectroscopy struggle to capture the formation and transformation of multicomponent crystals. The amount of solvent used during the crystal formation processes poses an obstacle for the sensitivity of these measurement methods. In addition, the design of some devices does not allow for online measurement methods. Furthermore, in cases of fast formation/transformation processes it can be difficult to obtain data with sufficient resolution to interpret the results. A flow-through glass capillary for X-ray measurements was recently introduced (Rohlíček *et al.*, 2020 ▸) to overcome some of the above mentioned limitations. The main advantage of this setup is that the transformation period can be captured by methods commonly used to characterize crystalline samples, such as X-ray diffraction. In this work, the flow-through capillary was used to study the process of transformation of the pharmaceutical ingredient ibrutinib (FDA, 2018 ▸; Rozovski *et al.*, 2014 ▸; Young & Staudt, 2014 ▸; Puig de la Bellacasa *et al.*, 2014 ▸; Veeraraghavan *et al.*, 2015 ▸) into solvates with a series of halogenated benzenes (Fig. 1 ▸) [fluoro­benzene (FBZ), chloro­benzene (CBZ), bromo­benzene (BBZ) and iodo­benzene (IBZ)]. This series was explored due to the similarity in chemical nature of the guest molecules and their ability to form multicomponent crystal solvates.

## 2. Methods and materials

The non-solvated form of Ibrutinib C (Zvoníček *et al.*, 2017 ▸, 2018 ▸; Purro & David, 2013 ▸) was provided by Zentiva k.s. and used as a starting material in all experiments performed. Solvents were obtained from various suppliers and used as received.

### 2.1. Single-crystal X-ray diffraction

SCXRD of BBZ and IBZ ibrutinib solvates was performed at 95 K using a SuperNova diffractometer with a micro-focus sealed tube, mirror-collimated Cu *K*α radiation (λ = 1.54184 Å) and CCD detector Atlas S2. The X-ray diffraction measurements of the structures of apremilast with phthalic acid and *o*-xylene were carried out at 120 K on an Xcalibur, Gemini ultra diffractometer using Cu *K*α radiation (λ = 1.54178 Å) from a fine-focus sealed X-ray tube with a graphite monochromator and CCD detector Atlas S2.

The data reduction and absorption correction were carried out using the *CrysAlisPro* software (Rigaku Oxford Diffraction, 2019 ▸). The structure was solved by charge-flipping methods using the *Superflip* software (Palatinus & Chapuis, 2007 ▸) and refined by full matrix least squares on the *F* squared value using the *Crystals* software (Betteridge *et al.*, 2003 ▸). *MCE* software (Rohlíček & Hušák, 2007 ▸) was used for visualization of residual electron density maps. According to common practice, the hydrogen atoms attached to carbon atoms were placed geometrically with *U*_iso_(H) in the range 1.2–1.5 *U*_eq_ of the parent atom (C). The structures have been deposited in the Cambridge Structural Database (CCDC nos. 2166306, 2166307 and 2164831).

### 2.2. Powder X-ray diffraction

Diffraction patterns of the sample in the flow-through capillary were collected with the powder diffractometer device Empyrean of PANalytical, with a Cu *K*α X-ray beam (λ = 1.542 Å, focusing mirror, voltage: 45 kV, current: 40 mA). The fast scans were measured in the range 5–9° 2θ with a step size of 0.013° 2θ and an overall measurement time of 5 min. The measurement for the structure determination was performed on a flat sample at 3–80° 2θ with a step size of 0.013° 2θ and an overall measurement time of 20 h.

The diffraction patterns of resulting solid samples from competitive slurries were collected with the Bruker AXS D8 powder diffractometer, a Cu *K*α X-ray beam (λ = 1.7903 Å), 5–40° 2θ measured range, 34 kV excitation voltage, 30 mA anodic current and 0.0196° 2θ step size. The measurement was performed on a flat sample with an area/thickness ratio equal to 10/0.5 mm. The *HighScore Plus* (Degen *et al.*, 2014 ▸) software was used to process the diffraction patterns.

### 2.3. Differential scanning calorimetry

Samples for differential scanning calorimetry (DSC) measurements were weighed in an aluminium pan (5–10 mg). The pan was covered and the measurement was carried out under a nitro­gen gas flow of 50 ml min^−1^. All the measurements were performed on the DSC 822^e^, Mettler Toledo instrument. The range of investigated temperatures was 0 to 300°C at a heating rate of 10°C min^−1^ (amplitude = 0.8°C, period = 60 s).

### 2.4. Thermogravimetric analysis

Thermogravimetric analysis (TGA) was performed on a TG 209, Netzsch instrument. Approximately 10 mg of the sample was weighed into a ceramic pan and measured under a nitro­gen atmosphere. The TGA measurement was performed in the approximate temperature range 20–300°C. A heating rate of 10°C min^−1^ was used in all experiments.

### 2.5. Raman spectroscopy

Samples for Raman spectroscopy were measured in HPLC glass vials in an FT-Raman RFS100/S spectrometer device with a germanium detector (Bruker Optics, Germany). The wavelength of the Nd:YAG laser was 1064 nm. The measurement range was 4000 to 0 cm^−1^, with a spectral resolution of 4.0 cm^−1^. Data were obtained at either 64 or 128 accumulations of the measured spectra. The software *OPUS* and *OMNIC* were used to process the Raman spectra.

### 2.6. Competitive slurry

All combinations of solvents were mixed in equimolar ratios in HPLC vials. Approximately 150 µl of each solvent was used. A mass of 100 mg of ibrutinib C was subsequently mixed with all possible combinations of the solvents. Vials were placed in a ThermoMixer C shaker at room temperature and mixed at 600 rev min^−1^. Vials were slurried for 48 h. The slurry was filtered and dried for 24 h at 40°C and 150 mbar. The prepared samples were analyzed using PXRD to confirm solvate structure formation and NMR to determine the ratio of solvents in the solid samples.

### 2.7. Nuclear magnetic resonance

Nuclear magnetic resonance (NMR) spectra were obtained using a Bruker Advance 500 (Bruker Biospin, Germany) at 500.13 MHz with a 5 mm Prodigy probe. Liquid NMR experiments were performed in di­methyl­sulfoxide or deuterated. The measurement temperature was 298 K.

### 2.8. Interaction energy calculations

Several approaches were chosen to estimate the interaction energy within the crystal structures. Solved solvate structures and ibrutinib polymorph C were optimized in the *CASTEP* program (Clark *et al.*, 2005 ▸). Final enthalpies of all optimized structures were then used to obtain the interaction energies of the solvates: *E*_int_ = *E*_solvate_ − (*E*_ibrutinib_ + *E*_solvent_). A different approach was based on isolated molecules and unit cells using the *Gaussian16* software (Frisch, *et al.*, 2016 ▸). The interaction energy was obtained with 2*E*_int_ = *E*_solvate_ − (*E*_ibrutinib_ + *E*_solvent_). Finally, crystal elongation energies were estimated using structures containing optimized unit cells in different directions (*i.e.* 2 × 1 × 1, 1 × 2 × 1, 1 × 1 × 2). Crystal elongation energies in each direction were established according to the formula *E*_elongation_ = *E*_2 × 1 × 1_ − 2*E*_1 × 1 × 1_.

## 3. Results and discussion

The ibrutinib solvates used to explore solid-state transformations were firstly characterized from a crystallographic point of view. The mechanism and kinetics of the transformations were studied using X-ray diffraction and complemented by thermodynamic experiments. Furthermore, the interaction energies of the crystal structures were calculated and compared with the experimental results.

### 3.1. Crystallography

To obtain insight into the crystal packing and intermolecular interactions, single crystals of all solvates were analyzed by SCXRD and their structures were solved. Crystal structures of the CBZ and FBZ solvates have been already reported in the literature as well as their comprehensive crystallographic analyses (Zvoníček *et al.*, 2017 ▸; Vasilopoulos *et al.*, 2022 ▸; Rohlíček *et al.*, 2020 ▸). The packing similarities of the solvate crystal structures were explored using *CrystalCMP* (Rohlíček *et al.*, 2016 ▸) and further crystallographic details of the structures are listed in the supporting information.

**Figure 2:**
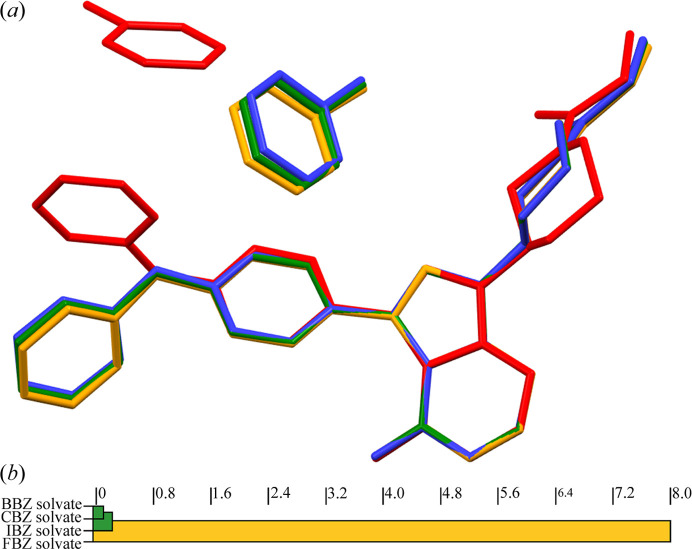
(*a*) Comparison of the solvate crystal structures (red – FBZ solvate, green – CBZ solvate, blue – BBZ solvate, orange – IBZ solvate). (*b*) Packing similarity dendrogram generated by *CrystalCMP*.

All four solvates crystallize in the triclinic system in the space group *P*1, with two molecules of ibrutinib and two molecules of the respective solvent in the unit cell. When compared from the point of view of molecular packing and unit-cell parameters, three of the solvates are isostructural (CBZ, BBZ and IBZ) and one is different (FBZ). The unit cells of the isostructural solvates (CBZ, BBZ and IBZ) contain two molecules of ibrutinib and two molecules of the respective solvent. Their cell parameters are very similar: *a* ≃ 11 Å, *b* ≃ 12 Å, *c* ≃ 12 Å, α ≃ 80°, β ≃ 69°, γ ≃ 69°. The unit-cell parameters of the FBZ solvate are *a* ≃ 9.6 Å, *b* ≃ 11.1 Å, *c* ≃ 14.2 Å, α ≃ 73.2°, β ≃ 82.1°, γ ≃ 66.0°. Fig. 2 ▸(*a*) shows the overlay of the conformations of ibrutinib and the positions of the solvent molecule. The FBZ solvate crystal structure differs due to the rotation of the outer parts of the ibrutinib molecule. FBZ also occupies a different spot in the FBZ solvate structure compared with the other solvents.

Fig. 2 ▸(*b*) shows the molecular packing similarity tree diagram calculated by *CrystalCMP*. It indicates that the molecular packing of ibrutinib molecules is almost identical in the three isostructural solvates. CBZ, BBZ and IBZ form isostructural cavity solvates with ibrutinib, whereas FBZ forms a channel solvate (see Fig. S4 of the supporting information).

### 3.2. Mechanism and kinetics of transformation

**Figure 3:**
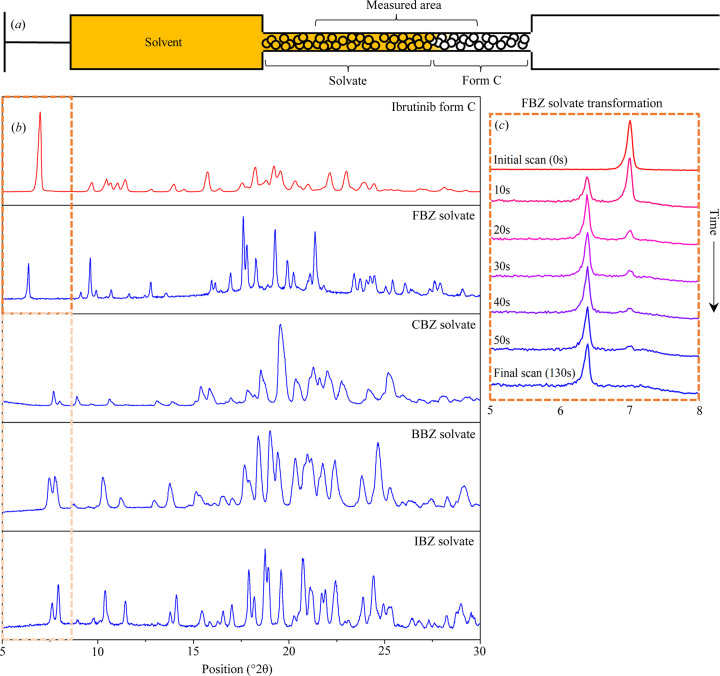
(*a*) Scheme of the glass flow-through capillary. (*b*) Diffraction patterns of ibrutinib polymorph C and of the prepared ibrutinib solvates. (*c*) Time series of diffraction patterns observed during the transformation of ibrutinib C to the FBZ solvate.

The transformations from ibrutinib C to halogen benzene solvates were studied using a flow-through capillary. We filled the glass capillary with ibrutinib C and mounted it in the PXRD device. We then initiated the measurement sequence of PXRD patterns, and the solvent was pumped through the glass capillary. Polymorph C of ibrutinib transformed into the solvated form following contact of the solid and liquid phases. We obtained a time series of diffraction patterns to monitor the phase transition. Fig. 3 ▸(*a*) depicts a scheme of the glass capillary.

Due to isostructural crystal packing, there is a certain degree of similarity in the diffraction patterns of CBZ, BBZ and IBZ solvates [see Fig. 3 ▸(*b*)]. However, the diffraction patterns of ibrutinib C and the ibrutinib solvates are significantly different between 5 and 9° [Fig. 3 ▸(*a*)]. The fact that each form has well defined, intensive peaks (at approximately 6.4° for the FBZ solvate and 7° for ibrutinib C) in this region helps to easily distinguish their diffraction patterns. The diffraction patterns of the CBZ, BBZ and IBZ solvates in this region also exhibit two characteristic peaks at approximately 8°. Therefore, we conveniently selected the region of 5–9° to monitor the transformations in all experiments performed. A certain degree of similarity in the diffraction patterns of CBZ, BBZ and IBZ can be observed owing to isostructural crystal packing. Fig. 3 ▸(*c*) shows the time evolution of the diffraction patterns after filling the flow-through capillary with FBZ. The changes in diffraction patterns reflect the transformation of non-solvated polymorph C to the FBZ solvate. Only ibrutinib C is present in the sample at the start of the measurement (the only peak present is at 7°). The gradual transformation of the sample is reflected by the extinction of the ibrutinib peak at ∼7° and progressive evolution of the FBZ solvate peak at 6.4°. The sample underwent full transformation to the FBZ solvate after complete extinction of the ibrutinib C peak at 7°. This entire transformation process takes approximately 130 s. We can describe the FBZ transformation as a direct (without any intermediate phases) and fast transformation that results in a channel solvate structure

A larger range (5–30°) was measured only before and after completion of the transformation. We measured only several degrees (5–9°) to minimize the required measurement time, which allows more frequent measurements during the transformation and therefore results in more accurate observation of the transformation process. Nevertheless, an optimal balance needs to be found between the quality of the measured data and the time required for the measurement. The transformation of ibrutinib C to the FBZ solvate was measured at 10 s intervals. In some cases, the 10 s time resolution between measurements was too low. Therefore, the diffraction patterns were then measured every 3 s, which resulted in more frequent, but also noisy data. Measuring the entire diffraction pattern would not provide any useful information, because the transformation would finish much faster than the measurement of a single diffraction pattern. However, the entire diffraction pattern was measured before and after the transformation process to confirm the complete transformation of the sample.

**Figure 4:**
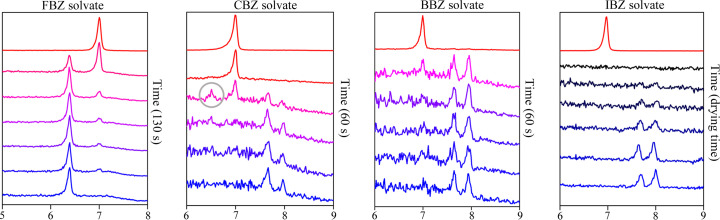
Transformation of ibrutinib form C to all four solvates illustrated by the evolution of diffraction patterns.

We performed the same procedure and evaluation for the three remaining solvents. The time evolution of the diffraction patterns for all four transformations is shown in Fig. 4 ▸. The observed noise in a number of the diffraction patterns can be attributed to the very short measurement time required to capture the fast transformation. It is interesting that the time evolution of diffraction patterns is different for each of the transformations, despite the similarity in chemical nature of the solvents and the similarity in the crystal packing (with the exception of the FBZ solvate).

**Figure 5:**
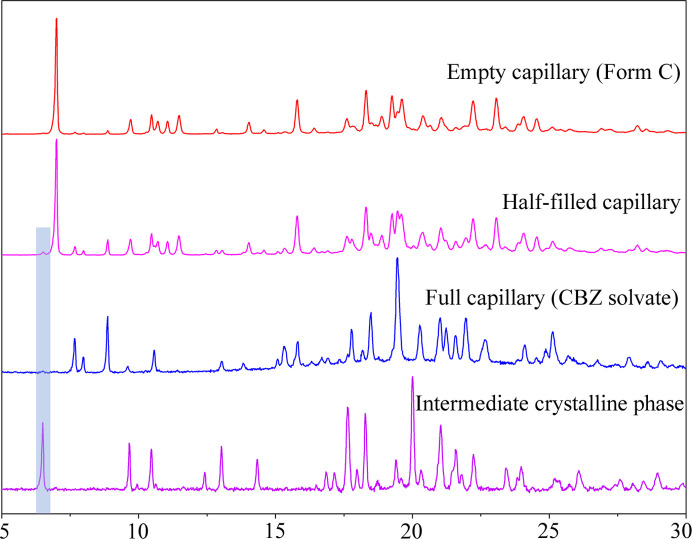
Measured diffraction patterns of the empty capillary (ibrutinib form C), half-filled capillary (ibrutinib form C + CBZ solvate + intermediate crystalline phase), full capillary (CBZ solvate) and intermediate crystalline phase.

As discussed above, the FBZ solvate transformation is fast (approximately 130 s) and direct. In contrast, the transformation of ibrutinib C to the CBZ solvate is more complex as indicated by the PXRD measurement of the transformation. A new peak at 6.5° appears in the diffraction patterns during the CBZ solvate transformation. This peak (marked by a gray circle in Fig. 4 ▸) does not correspond to ibrutinib C, or any other ibrutinib polymorph, nor the CBZ solvate. This indicates the formation of a new crystalline phase during the transformation period. The low-intensity and rapid extinction of the peak once we filled the capillary with CBZ led us to the conclusion that an intermediate crystalline form has a vital role in the formation of the CBZ solvate. This peak formed and was consistently present in experiments where only half of the capillary was filled with CBZ (PXRD measured both dry ibrutinib, wet ibrutinib and the interface between the two). Therefore, we assumed that the new intermediate phase forms at the interface of the solvated and non-solvated sample. After the interface is supplied with more solvent, the intermediate phase transforms into the CBZ solvate structure and the peak at 6.5° is no longer present. We successfully isolated the intermediate phase with further experiments (a detailed description is provided in the supporting information) and measured its diffraction pattern, which confirms the formation of the intermediate crystalline phase during the CBZ solvent transformation (Fig. 5 ▸).

The peak with low intensity at 6.5° in the half-filled capillary experiment corresponds well to the peak at 6.5° of the intermediate crystalline phase (highlighted by the gray area in Fig. 5 ▸). Only a very small amount of the intermediate phase is present in the half-filled capillary experiment, located at the interface of the solvated and non-solvated samples. This causes very low intensity of the peak, since the interface is only a small part of the measured area. The intermediate crystalline phase was further studied, and its crystal structure was successfully solved from PXRD data (CCDC no. 2164831). We discovered that the intermediate phase contains only one molecule of CBZ and two molecules of ibrutinib in the asymmetric unit. It is a CBZ hemisolvate with unit-cell parameters of *a* ≃ 14.0 Å, *b* ≃ 10.2 Å, *c* ≃ 10.4 Å, α ≃ 116.4°, β ≃ 85.6°, γ ≃ 79.3°, and the space group of the hemisolvate was determined to be *P*1.

This is in line with the assumption of the intermediate phase formation at the interface. A hemisolvate is formed at the interface, where there is an insufficient amount of CBZ molecules. After additional CBZ is supplied, the transformation continues further from the CBZ hemisolvate form to the final CBZ solvate (1:1) form.

Overall, the transformation of the CBZ solvate is more complex than in the case of the FBZ solvate. It is a fast transformation with a crystalline intermediate phase, where the first step is the transformation of ibrutinib C to the crystalline intermediate CBZ hemisolvate, which in a very short time can transform further into the CBZ (1:1) solvate after additional supply of CBZ.

The BBZ solvate transformation is much simpler (see Fig. 4 ▸). It is a fast and direct transformation, as in the case of the FBZ transformation. Nevertheless, the difference between the BBZ and FBZ transformations is in their final forms. The BBZ solvate is a cavity solvate with a different crystal structure compared with the FBZ channel solvate.

The mechanism of the IBZ solvate transformation is vastly different compared with the rest of the solvates. The sample becomes amorphous after the ibrutinib C makes contact with IBZ. This is confirmed by the diffraction patterns with no peaks. As long as the sample was in contact with the solvent, it remained amorphous. Visual inspection confirmed that the sample had not dissolved. The peaks in the diffraction pattern of the IBZ solvate began to evolve after the sample started to dry out and the excess IBZ evaporated. The evolution of the peaks, and the transformation itself, progressed continuously as the sample dried. The sample completely transformed after all the excess solvent evaporated. The sample changes to the amorphous form instantly on contact with the solvent. Therefore, the rate of transformation is entirely dependent on the slower phenomenon, the rate of drying.

Thus, we observed four different mechanisms of the transformation: FBZ – fast and direct transformation to the channel solvate structure, CBZ – fast transformation with a crystalline intermediate phase resulting in the cavity solvate structure, BBZ – fast and direct transformation to the cavity solvate structure, IBZ – fast transformation with an amorphous intermediate phase resulting in the cavity solvate structure. We compared experimental diffraction patterns of the samples after transformation with patterns generated from obtained crystal structures to ensure the consistency of the transformation process (Fig. S2).

### 3.3. Thermodynamics

We investigated the thermodynamic preference of the solvate structure formation to complement the results of the solvate transformations. For this purpose, we performed competitive slurry experiments. First, we created all possible pairs of our solvents and mixed them in the same molar ratios. Thus, as an example, a binary mixture of FBZ and CBZ in the molar ratio 1:1 was prepared. Analogously, all possible triplets and quartets of FBZ, CBZ, BBZ and IBZ were mixed in equimolar ratios. All prepared combinations of two, three and four solvents were slurred with ibrutinib C. Solvate structure formation was confirmed by PXRD and the content of each solvent in the solid sample was examined by liquid nuclear magnetic resonance spectroscopy (^1^H NMR). Table 1 ▸ lists the solvent contents in the solid samples for all competitive slurry experiments. We expected that the strongest-binding solvent in the solvate structures would be the most abundant in the final solvate sample. For example, a binary mixture of IBZ and FBZ (sample J in Table 1 ▸) contains almost exclusively IBZ solvent in the resulting solvate structure. This suggests stronger interactions of IBZ molecules in the solvate structure compared with FBZ molecules.

The sum of the analyzed solvent content is not always equal to one, despite following identical experimental procedure. In some cases, it was difficult to accurately evaluate the ^l^H NMR spectra, due to many overlapping bands, but the phenomenon of the preferential solvate formation is clear. Different rates of solvent drying from a solid sample may also contribute to the imperfect molar ratio. For instance, sample A is a mixture of all four solvents but the total content is higher than one. The observed ratios from that sample suggest the strongest preference for IBZ, while BBZ and CBZ behave similarly, and FBZ exhibits the lowest preference. We further explored the similarity of CBZ and BBZ in sample I, where in the binary system of the two solvents, BBZ had higher preference compared with CBZ. We observed the same trend of higher preference of BBZ over CBZ in triplet samples B and E. After a complete evaluation of Table 1 ▸, we suggest a preference sequence. Ibrutinib has the strongest preference to form the IBZ solvate and the least preferable is the FBZ channel solvate. The sequence of preference is as follows: IBZ > BBZ > CBZ > FBZ. This reflects the strength of interactions between ibrutinib and the respective solvents. We performed a set of DFT-based calculations to shed more light on the deep nature of interactions within the solvate structures which control their formation.

### 3.4. Calculations

**Figure 6:**
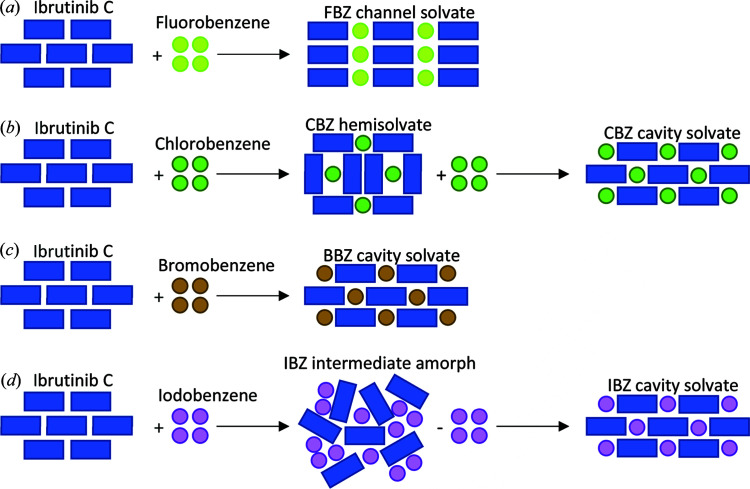
Different mechanisms of the transformation for FBZ, CBZ, BBZ and IBZ.

**Figure 7:**
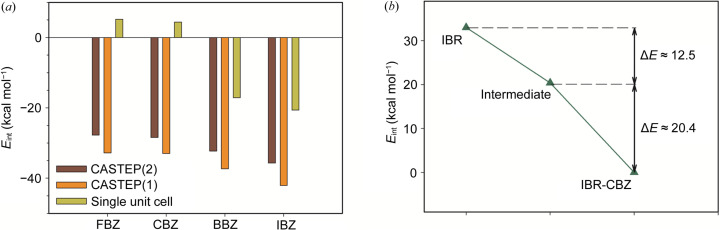
(*a*) *E*_int_ estimated using two *CASTEP*-based approaches and for the isolated system. (*b*) Relative *E*_int_ for the ibrutinib–CBZ solvate formation.

We applied two basic DFT-based approaches to explain the increasing tendency of ibrutinib to form a halogen benzene solvate with increasing halogen atomic number. Both approaches focused on estimations of interaction energies (*E*_int_) of a solvate unit cell associated with the formation of the cell from individual components. For more details, see the Methods in the supporting information. The first approach calculates *E*_int_ using the periodic conditions in *CASTEP* (Clark *et al.*, 2005 ▸), whereas the second treats a solvate or its components as isolated systems (*i.e.* as single unit cells or isolated molecules). To test the sensitivity of *E*_int_ (or trends in *E*_int_) to structural parameters of a solvent, we tested two modifications of the *CASTEP* approach. One variation [labeled *CASTEP*(2)] used the chosen unit cell of the solvent containing two molecules of solvent. In the second variation [labeled *CASTEP*(1)] we placed only one molecule of the solvent in the unit cell. The unit cell for a solvent was arbitrarily created from the corresponding solvate unit cell as described in the Methods. Fig. 6 ▸(*a*) graphically summarizes *E*_int_ estimated by all three methods. Table S2 of the supporting information then shows individual values. In general, a lower *E*_int_ means a stronger interaction among moieties in a solvate and hence the higher preference for its formation. We can see that all approaches reproduced the trend of increased preference of formation effectively (*i.e.* FBZ < CBZ < BBZ < IBZ) as the energy increased in the same series. Nevertheless, neglecting environmental effects in the isolated system approach is obviously too crude, as it estimated a positive *E*_int_ for FBZ and CBZ. This would suggest that they will not form, which does not agree with the experiment. Both *CASTEP* approaches correctly estimated negative *E*_int_ values for all solvates with increasing absolute values in the FBZ < CBZ < BBZ < IBZ series (Fig. 7 ▸). The *CASTEP*(1) approach provides a larger *E*_int_ by about ∼5.2 kcal mol^−1^. The decrease of absolute values in the *CASTEP*(2) approach can be attributed to the mutual interaction of two solvent molecules in the optimized unit cell. This leads to the lower energy of the solvent that results in slightly higher *E*_int_. Nevertheless, we cannot decide whether the mutual interaction of solvent molecules in pure solvent (according to our model) is not excessive. Therefore, our predictions have a more qualitative than quantitative value. Similarly, we also estimated *E*_int_ of the CBZ intermediate. Fig. 6 ▸(*b*) shows the values relative to the CBZ solvate, clearly showing the formation course: IBR → intermediate → solvate. Optimized parameters of unit cells for all solvates are gathered in Table S3.

To identify a possible source of interactions between IBR and solvents, we also calculated *E*_elongation_ for all solvates to predict the energy associated with elongation of a crystal in a certain direction. For computational details, see the Methods. Table S4 summarizes *E*_elongation_ estimated for all solvates at two theoretical levels. The lower level (PBE/3-21G) provides likely overestimated values, as *E*_elongation_ decreases with increased basis set. We can see that elongation in one particular direction applies to all solvents associated with substantially larger *E*_elongation_. This corresponds to elongation in the direction with the highest contribution of parallel-displaced π–π interactions. Different directions with the strongest *E*_elongation_ for different solvates can be attributed to different unit-cell orientations. We observe a slight increase of *E*_elongation_ in the FBZ < CBZ < BBZ series, which could indicate a slight increase of crystal strength in the same order. Energies for IBZ seem unreliably low due to the low-level of theory or the neglect of relativistic contributions.

Thermal behavior of the solvates is consistent with the results obtained from the competitive slurry experiment and the interaction energy calculations. The melting temperatures of the prepared solvates were evaluated and the IBZ solvate shows the highest melting temperature at 125°C. The melting temperature of the BBZ solvate was 110°C, the CBZ solvate was 96°C and the FBZ solvate was around 99°C. The calculated interaction strength as well as the preferred formation of the IBZ solvate correlate well with its higher thermal stability. The descending melting temperatures of BBZ and CBZ solvates follow equal logic. The FBZ solvate cannot be easily compared with the rest of the solvates since its crystal structure is different. Even though the descending order of melting temperatures cannot be considered direct proof of interaction strength, it provides additional insight and information about the solvate series.

## 4. Conclusions

The mechanism of solvate structure formation, thermodynamics and calculations form a coherent set of results. The calculated interaction energies of the solvent and ibrutinib molecules agree well with the increasing tendency of ibrutinib to form a halogen benzene solvate with increasing halogen atomic number, determined from competitive slurry experiments. The calculated interaction energy was lowest for the IBZ solvate, followed by the BBZ, CBZ and FBZ solvates which have increasingly higher interaction energies. The preference of solvate formation reflects the interaction energies well as the IBZ solvate forms more easily than the other solvates, followed by BBZ, CBZ and FBZ, which form in lower amounts, respectively, in the presence of other solvents. Further, we propose a hypothesis based on the observed results about how the interaction strength impacts the mechanism of solvate formation. During the formation of the IBZ solvate the sample becomes amorphous on contact with the IBZ solvent. We attribute this phenomenon to the strength of the IBZ and ibrutinib interaction (higher than the other solvates) which disrupts the crystalline structure of ibrutinib in excess IBZ. The crystalline order is restored after evaporation of the excess molecules of solvent and the cavity solvate is formed. In the case of the BBZ solvate, the interaction is weaker, and the mechanism of transformation is direct, without the amorphous intermediate phase. The resulting crystal structure is also the cavity solvate, which is isostructural with the IBZ solvate. The interaction strength of CBZ and ibrutinib is slightly weaker again. The mechanism of the CBZ solvate transformation requires a crystalline intermediate phase (hemi-CBZ solvate) that is more like the channel FBZ solvate, which has the weakest interaction strength. After a supply of additional CBZ the crystal structure transforms to the final cavity CBZ solvate. The FBZ solvate transforms directly, but the FBZ solvate is not isostructural with the rest of the solvates; it forms the channel solvate as opposed to a cavity solvate.

Overall, the system is fully characterized from the crystal structure and mechanism of transformation to the interaction energies and provides deep insight into the formation of ibrutinib solvates.

## 5. Related literature

The following references are cited in the supporting information: Macrae *et al.* (2020 ▸); Perdew *et al.* (1996 ▸); Grimme (2006 ▸); Monkhorst & Pack (1976 ▸).

## Supplementary Material

Crystal structure: contains datablock(s) global, bromo, iodo, chloro. DOI: 10.1107/S2052252523001197/lq5049sup1.cif

Structure factors: contains datablock(s) bromo. DOI: 10.1107/S2052252523001197/lq5049bromosup2.hkl

Structure factors: contains datablock(s) iodo. DOI: 10.1107/S2052252523001197/lq5049iodosup3.fcf

Supporting information, figures and tables. DOI: 10.1107/S2052252523001197/lq5049sup4.pdf

CCDC references: 2166306, 2166307, 2164831, 2164831, 2166307

## Figures and Tables

**Table 1:** Solvent content in slurry experiments (A: 4 solvents, B–E: 3 solvents, F–K: 2 solvents) as estimated by ^l^H NMR

	Solvent			
Ibrutinib C	FBZ	CBZ	BBZ	IBZ
A	0	0.3	0.33	0.5
B	–	0	0.3	0.5
C	0	–	0.33	0.72
D	0	0.26	–	0.85
E	0	0.33	0.65	–
F	–	–	0.4	0.69
G	–	0.2	–	0.85
H	0.07	–	–	0.9
I	–	0.35	0.75	–
J	0	–	0.98	–
K	0	0.4	–	–

## References

[bb1] Aakeröy, C. B. & Salmon, D. J. (2005). *CrystEngComm*, **7**, 439–448. 10.1039/b811322j PMC2748950 20046916

[bb2] Aguiar, A. J. & Zelmer, J. E. (1969). *J. Pharm. Sci.* **58**, 983–987. 10.1002/jps.2600580817 5344535

[bb3] Amrutha, S., Giri, L., SeethaLekshmi, S. & Varughese, S. (2020). *Cryst. Growth Des.* **20**, 5086–5096.

[bb4] Arlin, J.-B., Florence, A. J., Johnston, A., Kennedy, A. R., Miller, G. J. & Patterson, K. (2011). *Cryst. Growth Des.* **11**, 1318–1327.

[bb5] Betteridge, P. W., Carruthers, J. R., Cooper, R. I., Prout, K. & Watkin, D. J. (2003). *J. Appl. Cryst.* **36**, 1487.

[bb6] Billot, P., Hosek, P. & Perrin, M.-A. (2013). *Org. Process Res. Dev.* **17**, 505–511.

[bb7] Bolton, O., Simke, L. R., Pagoria, P. F. & Matzger, A. J. (2012). *Cryst. Growth Des.* **12**, 4311–4314.

[bb8] Clark, S. J., Segall, M. D., Pickard, C. J., Hasnip, P. J., Probert, M. I. J., Refson, K. & Payne, M. C. (2005). *Z. Kristallogr. Cryst. Mater.* **220**, 567–570.

[bb9] Collier, E. A., Davey, R. J., Black, S. N. & Roberts, R. J. (2006). *Acta Cryst.* B**62**, 498–505. 10.1107/S0108768106012018 16710070

[bb10] Dash, S. G. & Thakur, T. S. (2021). *Cryst. Growth Des.* **21**, 449–461.

[bb103] Degen, T., Sadki, M., Bron, E., König, U. & Nénert, G. (2014). *Powder Diffr.* **29**, S13–S18.

[bb11] Desiraju, G. R. (1995). *Angew. Chem. Int. Ed. Engl.* **34**, 2311–2327.

[bb12] FDA (2018). *FDA Drug Approval Package: Imbruvica (ibrutinib)*, https://www.accessdata.fda.gov/drugsatfda_docs/label/2018/210563s000lbl.pdf.

[bb101] Frisch, M. J., Trucks, G. W., Schlegel, H. B., Scuseria, G. E., Robb, M. A., Cheeseman, J. R., Scalmani, G., Barone, V., Petersson, G. A., Nakatsuji, H., Li, X., Caricato, M., Marenich, A. V., Bloino, J., Janesko, B. G., Gomperts, R., Mennucci, B., Hratchian, H. P., Ortiz, J. V., Izmaylov, A. F., Sonnenberg, J. L., Williams-Young, D., Ding, F., Lipparini, F., Egidi, F., Goings, J., Peng, B., Petrone, A., Henderson, T., Ranasinghe, D., Zakrzewski, V. G., Gao, J., Rega, N., Zheng, G., Liang, W., Hada, M., Ehara, M., Toyota, K., Fukuda, R., Hasegawa, J., Ishida, M., Nakajima, T., Honda, Y., Kitao, O., Nakai, H., Vreven, T., Throssell, K., Montgomery, J. A. Jr, Peralta, J. E., Ogliaro, F., Bearpark, M. J., Heyd, J. J., Brothers, E. N., Kudin, K. N., Staroverov, V. N., Keith, T. A., Kobayashi, R., Normand, J., Raghavachari, K., Rendell, A. P., Burant, J. C., Iyengar, S. S., Tomasi, J., Cossi, M., Millam, J. M., Klene, M., Adamo, C., Cammi, R., Ochterski, J. W., Martin, R. L., Morokuma, K., Farkas, O., Foresman, J. B. & Fox, D. J. (2016). *Gaussian16*, Revision B. 01, Gaussian, Inc., Wallingford CT.

[bb106] Grimme, S. (2006). *J. Comput. Chem.* **27**, 1787–1799. 10.1002/jcc.20495 16955487

[bb13] Issa, N., Karamertzanis, P. G., Welch, G. W. A. & Price, S. L. (2009). *Cryst. Growth Des.* **9**, 442–453.

[bb14] Karamertzanis, P. G., Kazantsev, A. V., Issa, N., Welch, G. W. A., Adjiman, C. S., Pantelides, C. C. & Price, S. L. (2009). *J. Chem. Theory Comput.* **5**, 1432–1448. 10.1021/ct8004326 26609729

[bb15] Kokubo, H., Morimoto, K., Ishida, T., Inoue, M. & Morisaka, K. (1987). *Int. J. Pharm.* **35**, 181–183. 10.1002/jps.2600721217 6663481

[bb104] Macrae, C. F., Sovago, I., Cottrell, S. J., Galek, P. T. A., McCabe, P., Pidcock, E., Platings, M., Shields, G. P., Stevens, J. S., Towler, M. & Wood, P. A. (2020). *J. Appl. Cryst.* **53**, 226–235. 10.1107/S1600576719014092 PMC6998782 32047413

[bb16] Matsuda, H., Osaki, K. & Nitta, I. (1958). *Bull. Chem. Soc. Jpn*, **31**, 611–620.

[bb17] Matsuda, Y., Akazawa, R., Teraoka, R. & Otsuka, M. (2011). *J. Pharm. Pharmacol.* **46**, 162–167. 10.1111/j.2042-7158.1994.tb03770.x 8027920

[bb107] Monkhorst, H. J. & Pack, J. D. (1976). *Phys. Rev. B*, **13**, 5188–5192.

[bb18] S. de Moraes, L., Edwards, D., Florence, A. J., Johnston, A., Johnston, B. F., Morrison, C. A. & Kennedy, A. R. (2017). *Cryst. Growth Des.* **17**, 3277–3286.

[bb19] Musumeci, D., Hunter, C. A., Prohens, R., Scuderi, S. & McCabe, J. F. (2011). *Chem. Sci.* **2**, 883–890.

[bb20] Nauha, E. & Nissinen, M. (2011). *J. Mol. Struct.* **1006**, 566–569.

[bb21] Purro, N., Smyth, M. S. Goldman E. & Wirth D. D. (2013). Patent US 10294232 B2.

[bb22] Palatinus, L. & Chapuis, G. (2007). *J. Appl. Cryst.* **40**, 786–790.

[bb23] Pandit, J. K., Gupta, S. K., Gode, K. D. & Mishra, B. (1984). *Int. J. Pharm.* **21**, 129–132.

[bb105] Perdew, J. P., Burke, K. & Ernzerhof, M. (1996). *Phys. Rev. Lett.*, **77**, 3865–3868. 10.1103/PhysRevLett.77.3865 10062328

[bb24] Price, S. L. (2014). *Chem. Soc. Rev.* **43**, 2098–2111.

[bb25] Puig de la Bellacasa, R., Roué, G., Balsas, P., Pérez-Galán, P., Teixidó, J., Colomer, D. & Borrell, J. I. (2014). *Eur. J. Med. Chem.* **86**, 664–675. 10.1016/j.ejmech.2014.09.018 25222877

[bb26] Reilly, A. M., Cooper, R. I., Adjiman, C. S., Bhattacharya, S., Boese, A. D., Brandenburg, J. G., Bygrave, P. J., Bylsma, R., Campbell, J. E., Car, R., Case, D. H., Chadha, R., Cole, J. C., Cosburn, K., Cuppen, H. M., Curtis, F., Day, G. M., DiStasio, R. A. Jr, Dzyabchenko, A., van Eijck, B. P., Elking, D. M., van den Ende, J. A., Facelli, J. C., Ferraro, M. B., Fusti-Molnar, L., Gatsiou, C.-A., Gee, T. S., de Gelder, R., Ghiringhelli, L. M., Goto, H., Grimme, S., Guo, R., Hofmann, D. W. M., Hoja, J., Hylton, R. K., Iuzzolino, L., Jankiewicz, W., de Jong, D. T., Kendrick, J., de Klerk, N. J. J., Ko, H.-Y., Kuleshova, L. N., Li, X., Lohani, S., Leusen, F. J. J., Lund, A. M., Lv, J., Ma, Y., Marom, N., Masunov, A. E., McCabe, P., McMahon, D. P., Meekes, H., Metz, M. P., Misquitta, A. J., Mohamed, S., Monserrat, B., Needs, R. J., Neumann, M. A., Nyman, J., Obata, S., Oberhofer, H., Oganov, A. R., Orendt, A. M., Pagola, G. I., Pantelides, C. C., Pickard, C. J., Podeszwa, R., Price, L. S., Price, S. L., Pulido, A., Read, M. G., Reuter, K., Schneider, E., Schober, C., Shields, G. P., Singh, P., Sugden, I. J., Szalewicz, K., Taylor, C. R., Tkatchenko, A., Tuckerman, M. E., Vacarro, F., Vasileiadis, M., Vazquez-Mayagoitia, A., Vogt, L., Wang, Y., Watson, R. E., de Wijs, G. A., Yang, J., Zhu, Q. & Groom, C. R. (2016). *Acta Cryst.* B**72**, 439–459.

[bb102] Rigaku Oxford Diffraction (2019). *CrysAlisPro.* Rigaku Oxford Diffraction, Yarnton, UK.

[bb27] Rohlíček, J. & Hušák, M. (2007). *J. Appl. Cryst.* **40**, 600–601.

[bb28] Rohlíček, J., Skořepová, E., Babor, M. & Čejka, J. (2016). *J. Appl. Cryst.* **49**, 2172–2183.

[bb29] Rohlíček, J., Zvoníček, V., Skořepová, E. & Šoóš, M. (2020). *Powder Diffr.* **35**, 160–165.

[bb30] Rozovski, U., Hazan-Halevy, I., Keating, M. J. & Estrov, Z. (2014). *Cancer Lett.* **352**, 4–14. 10.1016/j.canlet.2013.07.013 PMC3871981 23879961

[bb31] Schultheiss, N. & Newman, A. (2009). *Cryst. Growth Des.* **9**, 2950–2967. 10.1021/cg900129f PMC2690398 19503732

[bb32] Sládková, V., Skalická, T., Skořepová, E., Čejka, J., Eigner, V. & Kratochvíl, B. (2015). *CrystEngComm*, **17**, 4712–4721.

[bb33] Stanton, M. K. & Bak, A. (2008). *Cryst. Growth Des.* **8**, 3856–3862.

[bb34] Sun, C. & Grant, D. J. W. (2001). *Pharm. Res.* **18**, 274–280. 10.1023/a:1011038526805 11442264

[bb35] Suresh, K., Minkov, V. S., Namila, K. K., Derevyannikova, E., Losev, E., Nangia, A. & Boldyreva, E. V. (2015). *Cryst. Growth Des.* **15**, 3498–3510.

[bb36] Vasilopoulos, Y., Heyda, J., Rohlíček, J., Skořepová, E., Zvoníček, V. & Šoóš, M. (2022). *J. Phys. Chem. B*, **126**, 503–512. 10.1021/acs.jpcb.1c07655 34994565

[bb37] Veeraraghavan, S., Viswanadha, S., Thappali, S., Govindarajulu, B., Vakkalanka, S. & Rangasamy, M. (2015). *J. Pharm. Biomed. Anal.* **107**, 151–158. 10.1016/j.jpba.2014.11.041 25594893

[bb38] Wöhler, F. (1844). *Annalen Chem. Pharm.* **51**, 145–163.

[bb39] Young, R. M. & Staudt, L. M. (2014). *Cancer Cell*, **26**, 11–13. 10.1016/j.ccr.2014.06.023 PMC4199743 25026208

[bb40] Zvoníček, V., Skořepová, E., Dušek, M., Babor, M., Žvátora, P. & Šoóš, M. (2017). *Cryst. Growth Des.* **17**, 3116–3127.

[bb41] Zvoníček, V., Skořepová, E., Dušek, M., Žvátora, P. & Šoóš, M. (2018). *Cryst. Growth Des.* **18**, 1315–1326.

