# Supplementary material for: Formation of ibrutinib solvates: so similar, yet so different
Source: IUCrJ. 2023 Feb 24;10(Pt 2):210–9. doi: 10.1107/S2052252523001197 (PMC9980385; doi:10.1107/S2052252523001197)
Supplement: Supplementary file 4 [file m-10-00210-sup4.pdf]

IUCrJ

Volume 10 (2023)

Supporting information for article:

Formation of ibrutinib solvates: so similar, yet so different

Jan Jirát, Jan Rohlíček, Jakub Kaminský, Tomáš Jirkal, Luděk Ridvan, Eliška Skořepová, Vít Zvoníček, Michal Dušek and Miroslav Šoóš

The supporting information presents additional results, that support and clarify statements in the manuscript and detailed description of the setup of intermolecular calculations.

Crystallography

Details from structure solution from single crystals of ibrutinib solvates with bromobenzene and iodobenzene are listed in Table S1. Table S1 contains crystallography details about the crystalline intermediate phase, chlorobenzene hemisolvate, solved from powder X-ray diffraction data as well.

Table S1. Crystallography details and structure solution details.

Number	1	2	3
Guest molecule	Bromobenzene	Iodobenzene	Chlorobenzene hemisolvate
CCDC deposit number	2166306	2166307	2164831
Empirical formula	C25 H24 N6 O2, C6 H5 Br	C25 H24 N6 O2, C6 H5 I	2(C25 H24 N6 O2), C6 H5 Cl
Diffractometer	SuperNova	SuperNova	Empyrean of PANalytical
Wavelength (Å)	1.54180	1.54180	1.54059
mol. weight (g/mol)	597.50	644.50	993.55
Crystal system	Triclinic	Triclinic	Triclinic
Space group	P 1	P 1	P 1
a (Å)	11.0298(4)	11.0044(2)	14.0474(3)
b (Å)	11.9154(4)	11.9170(2)	10.21941(15)
c (Å)	11.9588(4)	12.15470(10)	10.37318(18)
α (°)	80.855(3)	79.7701(11)	116.4028(10)
β (°)	71.713(3)	71.2027(13)	85.6175(14)
γ (°)	68.106(3)	68.7521(15)	79.3096(9)
Volume (Å3)	1383.10(5)	1403.023(12)	1289.11(4)
Z	2	2	1
Dens.(calc.) (g/cm3)	1.435	1.526	1.280
Abs. coeff. (mm-1)	2.350	9.287	1.133
F(000)	616.0	652.0	522.0
Crystal size (mm3)	0.082x0.122x0.368	0.075x0.108x0.260	-
Crystal description	prism	prism	-
θ range (°)	3.897; 71.192	3.851; 74.829	3.007; 79.989
Refl. collected	17859	24897	-
Indep. reflections	9964 [Rint =0.021]	10634 [Rint=0.017]	-
Reflections obs.	9901	10563	-
Criterion for obs.	[I > 2.0σ(I)]	[I > 2.0σ(I)]	-
Completeness to θ (°)	0.995 to 69.056	0.996 to 71.088	-
Absorption corr.	multi-scan	multi-scan	none
Min. and max. transm.	0.744 and 0.825	0.345 and 0.498	-
Data / restraints / param.	9964 / 11 / 734	10634/109/734	-/193/268

Goodness-of-fit on F2	1.0048	1.0029	3.37
Fin. R indices [I>2sigma(I)]	R1=0.0344, wR2=0.0943	R1=0.0277, wR2=0.0695	-
R indices (all data)	R1=0.0346, wR2=0.0943	R1=0.0277, wR2=0.0697	Rp=0.018, Rwp=0.024
Fin. diff. ρmax (e– /Å-3)	0.84 and -0.66	1.56 and -1.01	0.17 and -0.18
Temperature of measurement (K)	95	95	293

It is important to emphasize that similar form to the hemisolvate chlorobenzene structure that we observed from powder solution was described in a scientific literature(Zvoníček *et al.*, 2017). The intermediate chlorobenzene phase is isostructural with the *m*-xylene solvate. Therefore, such an arrangement of CBZ and ibrutinib molecules with insufficient amount of CBZ solvent is entirely possible.

The asymmetric unit of the solvates (FBZ, CBZ, BBZ and IBZ) contains two molecules of ibrutinib and two molecules of respective solvent. All the molecules of the asymmetric unit are overlayed in Figure S1 in comparison with Figure 2 in the manuscript where only one of each molecule is displayed to improve clarity of the figure in manuscript.

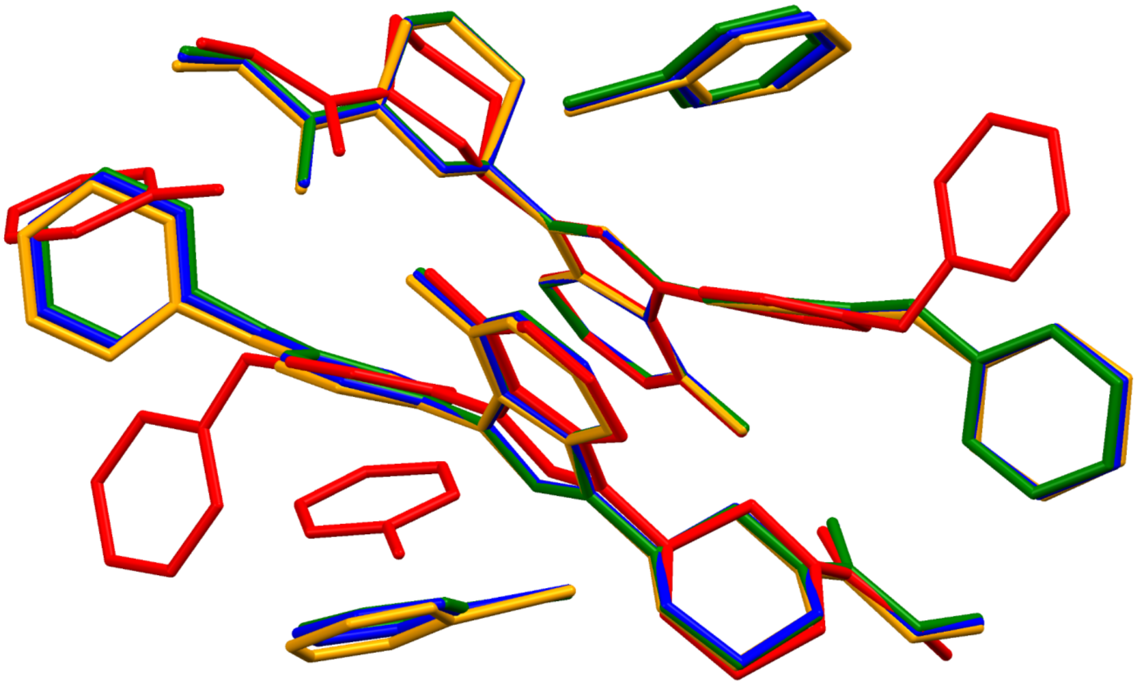

Figure S1. Overlay of all molecules in the asymmetric unit of the solvates (red; FBZ solvate, green; CBZ solvate, blue; BBZ solvate, orange; IBZ solvate).

Comparison of XRPD patterns

XRPD patterns calculated from crystal structures were compared with pattern obtained from real samples to ensure complete transformation of the samples to solvated forms.

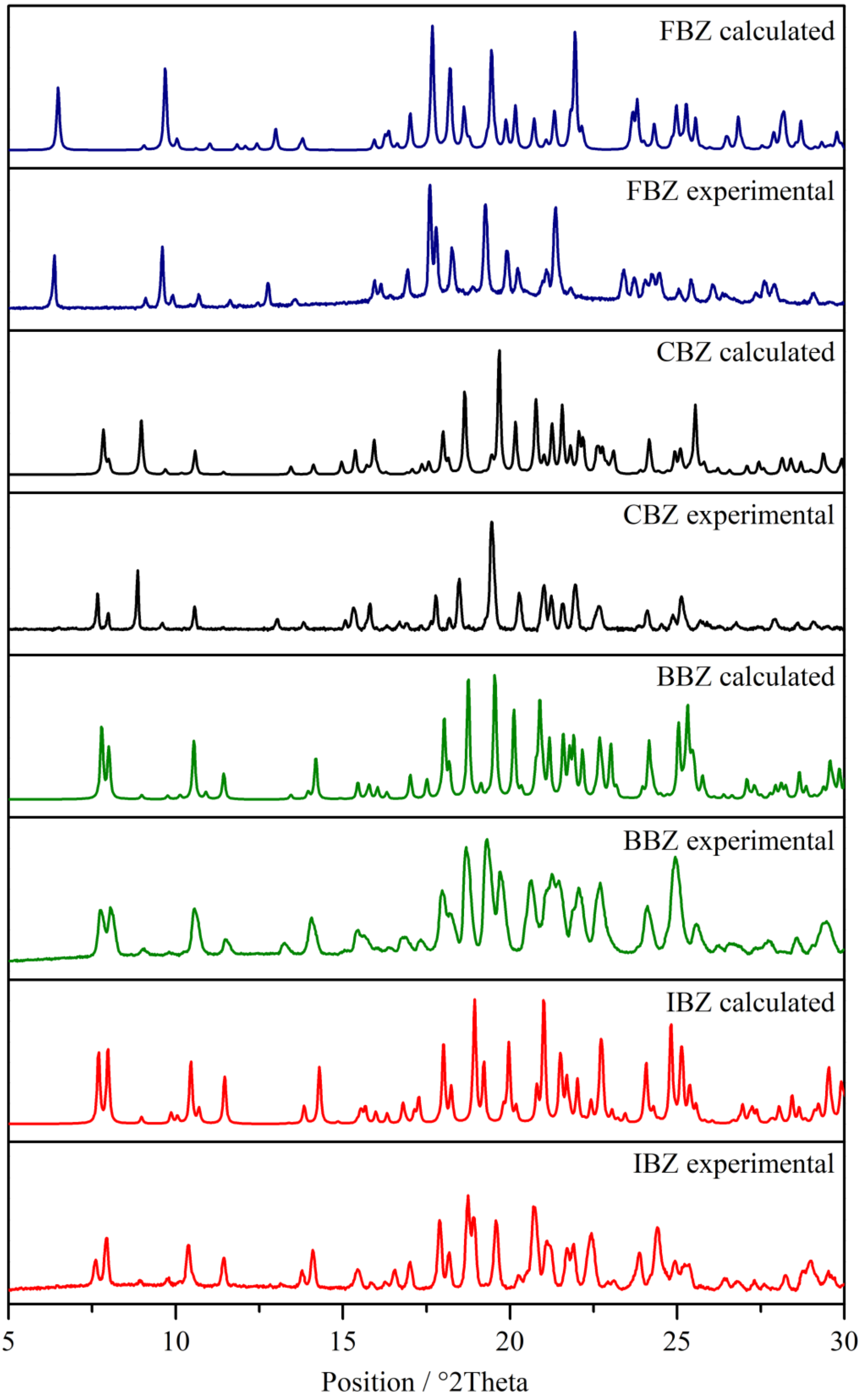

Figure S2. Comparison of XRPD patterns from solid samples and pattern calculated from solved crystal structures.

Raman spectroscopy

Raman spectra of the new forms were collected to complement the solid-state analysis.

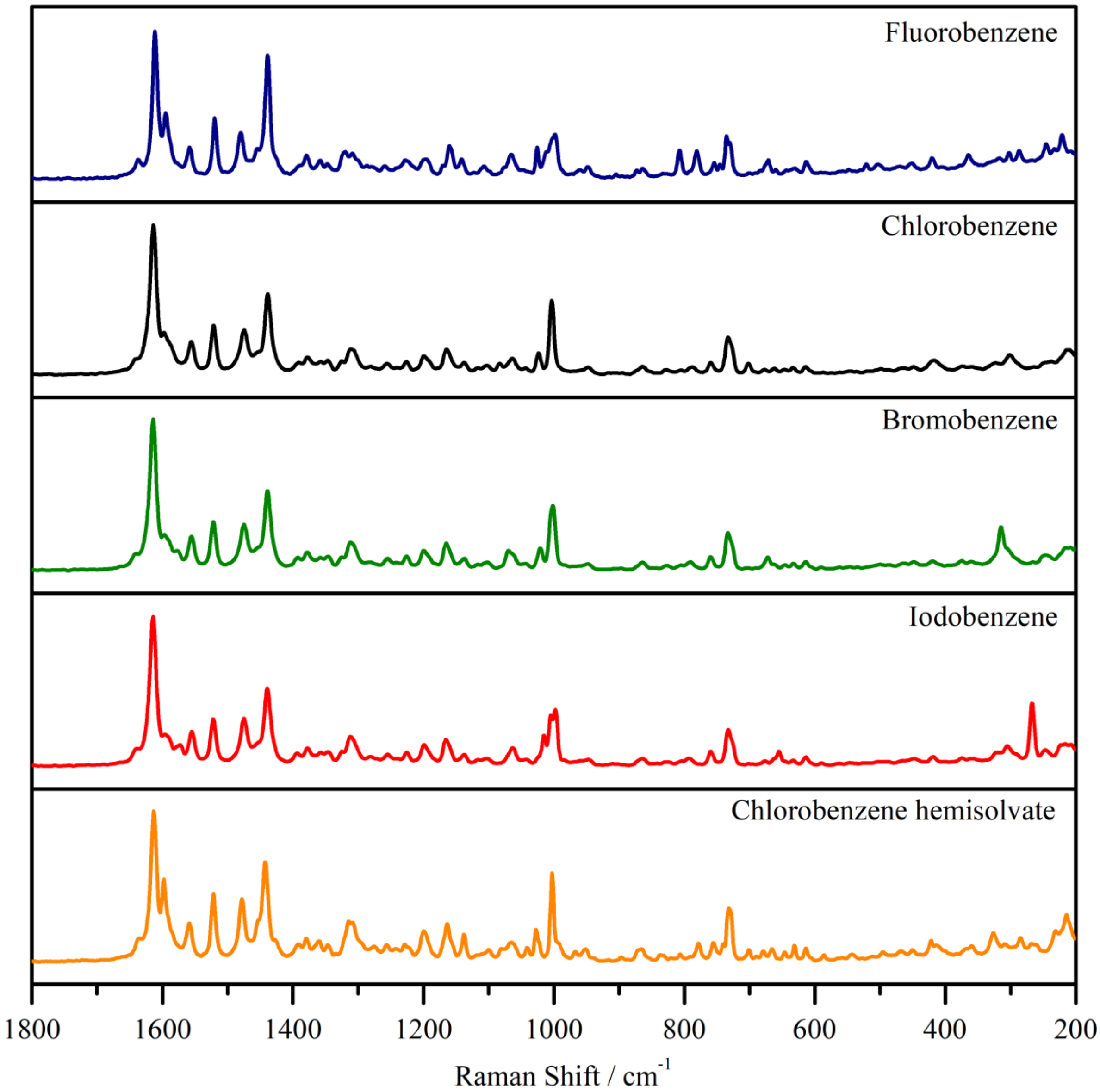

Figure S3. Raman spectra of prepared solid forms.

Channel and cavity solvate structures

Channel or cavity solvate structures refer to an empty space left when the solvent molecules are hidden in the solvate structures. Determining the voids (free space) in the structure provide additional insight about the process of desolvation. Voids were displayed in crystallography software Mercury(Macrae *et al.*, 2020). Default setting was used at the probe radius of 1.2 Å and approx. grid spacing of 0.7 Å.

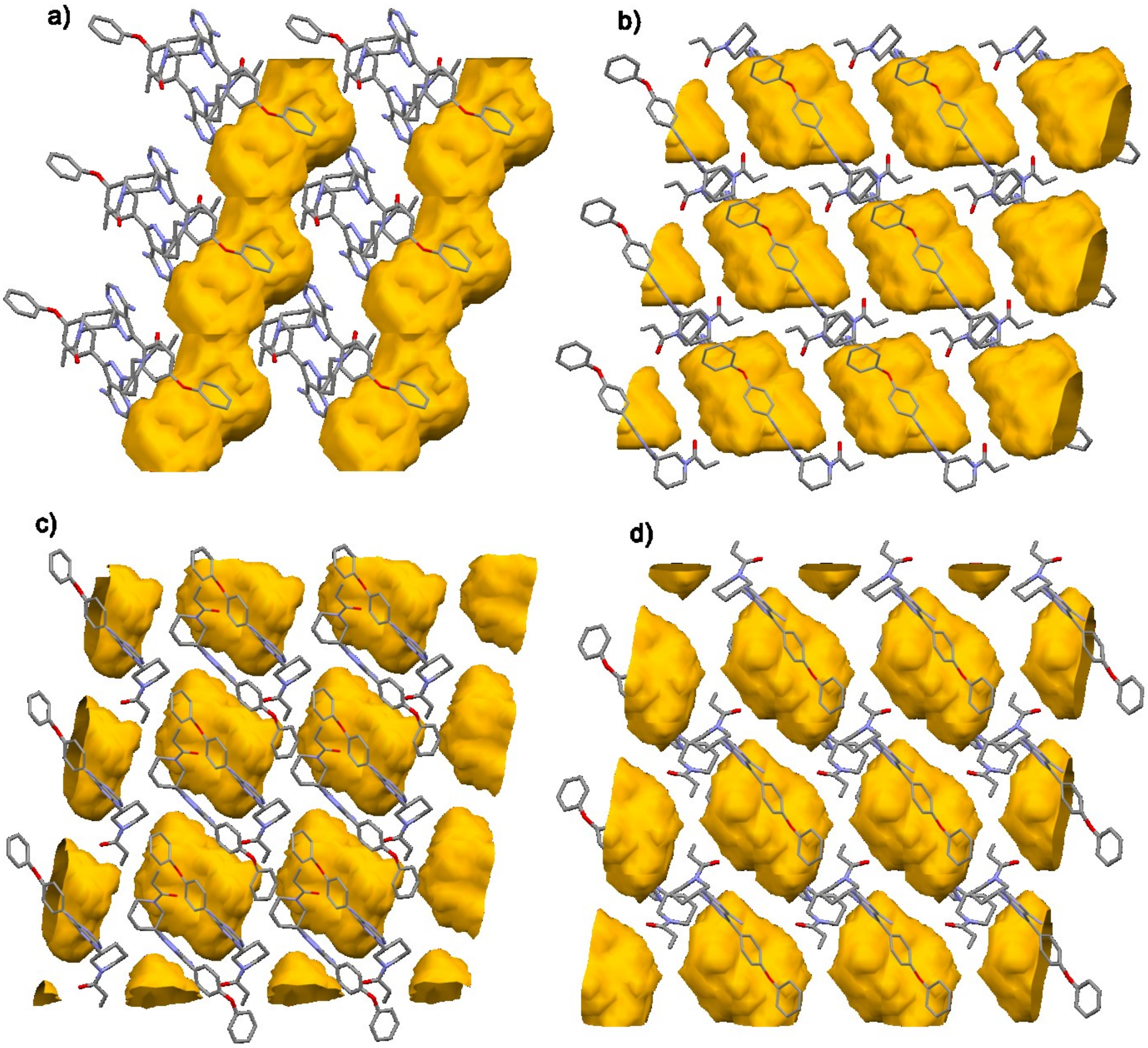

Figure S4. a) FBZ channel solvate, b) CBZ cavity solvate, c) BBZ cavity solvate, d) IBZ cavity solvate.

Interaction energy calculations

Several approaches were chosen to estimate the interaction energy within the crystal structures. Solved solvate structures and ibrutinib form C were optimized in CASTEP program(Clark *et al.*, 2005). Final enthalpies of all optimized structures were then used to obtain interaction energies of a solvate: $E_{int} = E_{solvate} - (E_{ibrutinib} + E_{solvent})$.Different approach was based on isolated molecules and unit-cells using Gaussian16 software. The interaction energy was obtained as $E_{int} = E_{solvate} - (E_{ibrutinib} + 2E_{solvent})$. Finally, crystal elongation energies were estimated using structures containing optimized unit cells in different directions (i.e. 2×1×1, 1×2×1, 1×1×2). Crystal elongation energies in each direction were established according to the formula: $E_{elongation} = E_{2\times1\times1} - 2E_{1\times1\times1}$. Interaction and elongation energies calculated by the above mentioned approaches are summarized in the Table S2, Table S3 and Table S4. The approaches are described in more detail in the methods section of supporting information.

Table S2. Calculated E_{int} (kcal/mol) as obtained by three different approaches (CASTEP, single unit-cell, and 2×2×2 unit-cells).

solvate	CASTEP(2)[a]	CASTEP(1)[b]	Single unit-cell[c]
FBZ	-27.74	-32.80	5.22
CBZ	-28.42	-32.95	4.42
BBZ	-32.27	-37.32	-17.14
IBZ	-35.72	-42.06	-20.61

[a] Two molecule of a solvent in the solvent unit-cell; [b] One molecule of a solvent in the solvent unit-cell; [c] ωB97XD/6-311++G**/LANL2DZ level of theory

Table S3. Optimized cell parameters (lengths in Å; angles in degrees) as obtained with CASTEP.

	FBZ	CBZ	BBZ	IBZ
a	9.6	11.1	11.0	11.0
b	11.1	11.9	11.9	11.9
c	14.2	11.9	12.0	12.2
α	73.2	81.3	80.9	79.8
β	82.1	67.8	71.7	71.2
γ	66.0	72.1	68.1	68.8

Table S4. Calculated $E_{elongation}$ (in kcal/mol) in different *a*, *b*, and *c* directions as depicted in Figure S10.

	PBE/3-21G			PBE/6-31+G**/LANL2DZ		
solvate	2×1×1	1×2×1	1×1×2	2×1×1	1×2×1	1×1×2
FBZ	-59.3	-11.8	-9.2	-43.8	-7.6	-5.8
CBZ	-64.2	-23.1	-20.5	-46.4	-15.2	-16.5
BBZ	-33.6	-62.3	-17.2	-23.0	-46.6	-12.7
IBZ	-22.8	-13.2	-27.1	-15.7	-10.8	-19.5

Differential scanning calorimetry (DSC)

The thermal behavior of prepared solvates was evaluated using DSC. Melting temperatures of solvates are as follows: FBZ ~ 99 °C, CBZ ~ 96 °C, BBZ ~ 110 °C, IBZ ~ 125 °C. The DSC pattern of FBZ solvate presents several peaks. The first peak at 99 °C reflects melting point of the FBZ solvate. We suspect that after the desolvation of the sample, it transforms to non-solvated ibrutinib form C. The form C melts at approx. 130 °C(Zvoníček *et al.*, 2018). It was reported in scientific literature that form C cannot be easily crystallized by conventional methods. It was prepared by desolvation of ibrutinib-methanol solvate(Zvoníček *et al.*, 2018). In the case of FBZ solvate we expect a similar transformation mechanism. Form C subsequently melts and recrystallizes into ibrutinib form A (melting point at 155 °C(Zvoníček *et al.*, 2018)). Melting temperature of the CBZ intermediate phase (CBZ hemisolvate) was measured and evaluated at 101 °C.

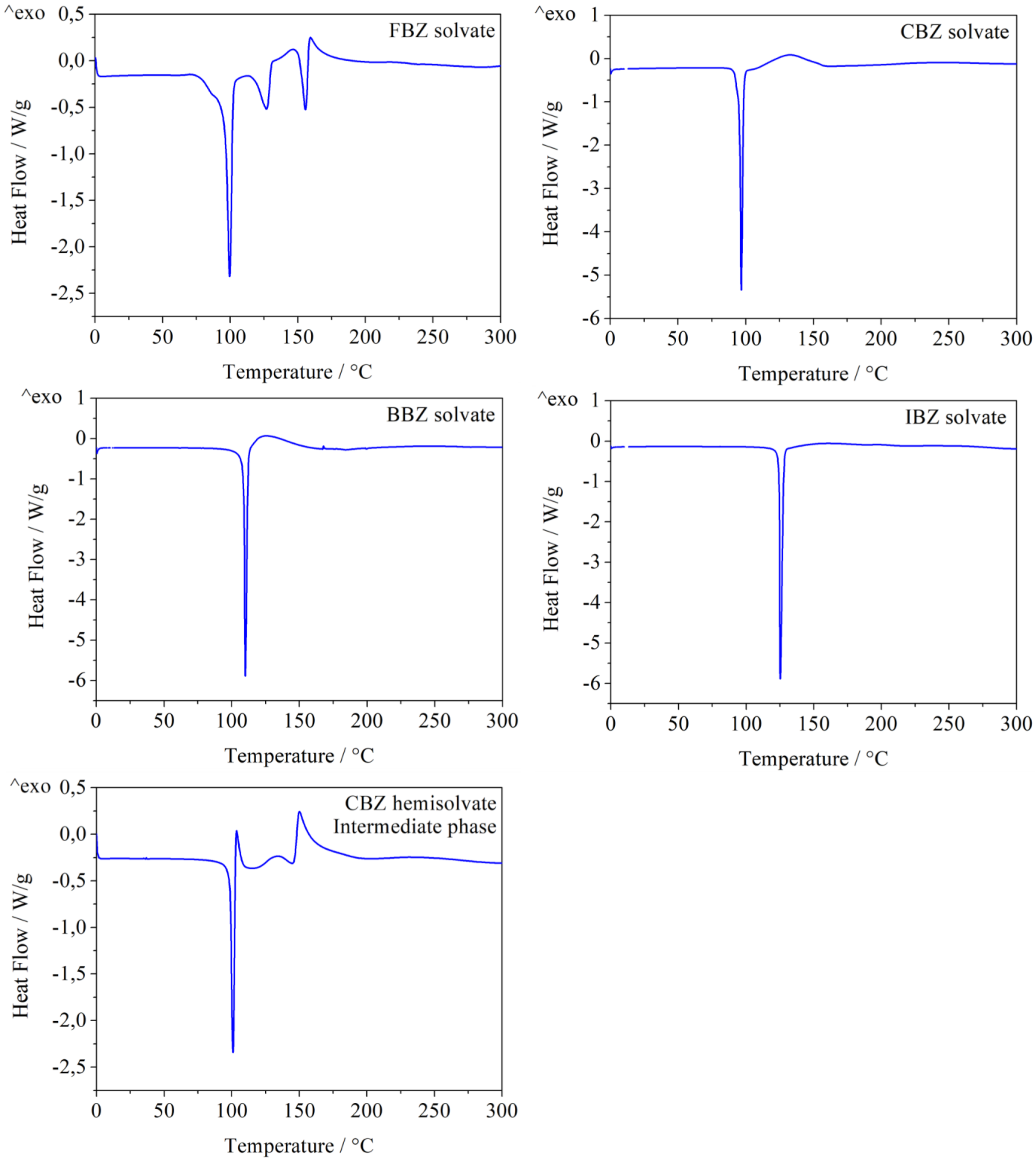

Figure S5. DSC curves of measured solvates.

Thermogravimetric analysis (TGA)

Thermogravimetric analysis was performed to provide additional information about the thermal behavior of the solvates. Theoretical amount of the solvent in the solvate sample was calculated from the solved crystal structures. The theoretical amount was subsequently compared with the amount evaporated from the solvate samples during the TGA measurement. In the case of CBZ solvate the theoretical amount that should evaporate is approx. 1/5 of the sample weight (20 %). After the TGA experiment was terminated the real weight loss was approx. 20 % (ratio of theoretical and real weight loss is approx. 1). The fact that the theoretical and real weight loss are very similar suggests that the

sample was properly dried. Free solvent completely evaporated during drying and the CBZ present in the crystalline structure did not evaporate. The ratio between theoretical and real weight loss was equal to 1 for BBZ and IBZ solvate as well. However, the FBZ solvate behaves differently. Despite equal drying conditions with the other solvates, the real weight loss during TGA measurement is much smaller compared to the theoretical weight loss. This phenomenon suggests much easier evaporation of the FBZ molecules integrated within the crystal structure. Easier evaporation of the molecules from the structure is most likely caused by the arrangement of the molecules in the crystal structure. The FBZ solvate forms channels, which allow the FBZ molecules to evaporate from the crystal structure more easily. The other solvates form cavities instead of channels. Lower boiling point of FBZ also contributes to easier evaporation of the solvent.

The different rates of drying from the samples is probably the main reason for imperfect molar ratios in the competitive slurry experiments discussed in the manuscript.

Table S5. Summarizes the ratio between theoretical and real weight loss during the thermogravimetric analysis.

Solvate	Theoretical solvent amount [%]	Real weight loss [%]	Real / theoretical
Iodobenzene	31.7	32.7	1.03
Bromobenzene	26.3	26.7	1.02
Chlorobenzene	20.4	20.6	1.01
Fluorobenzene	17.9	15.9	0.89

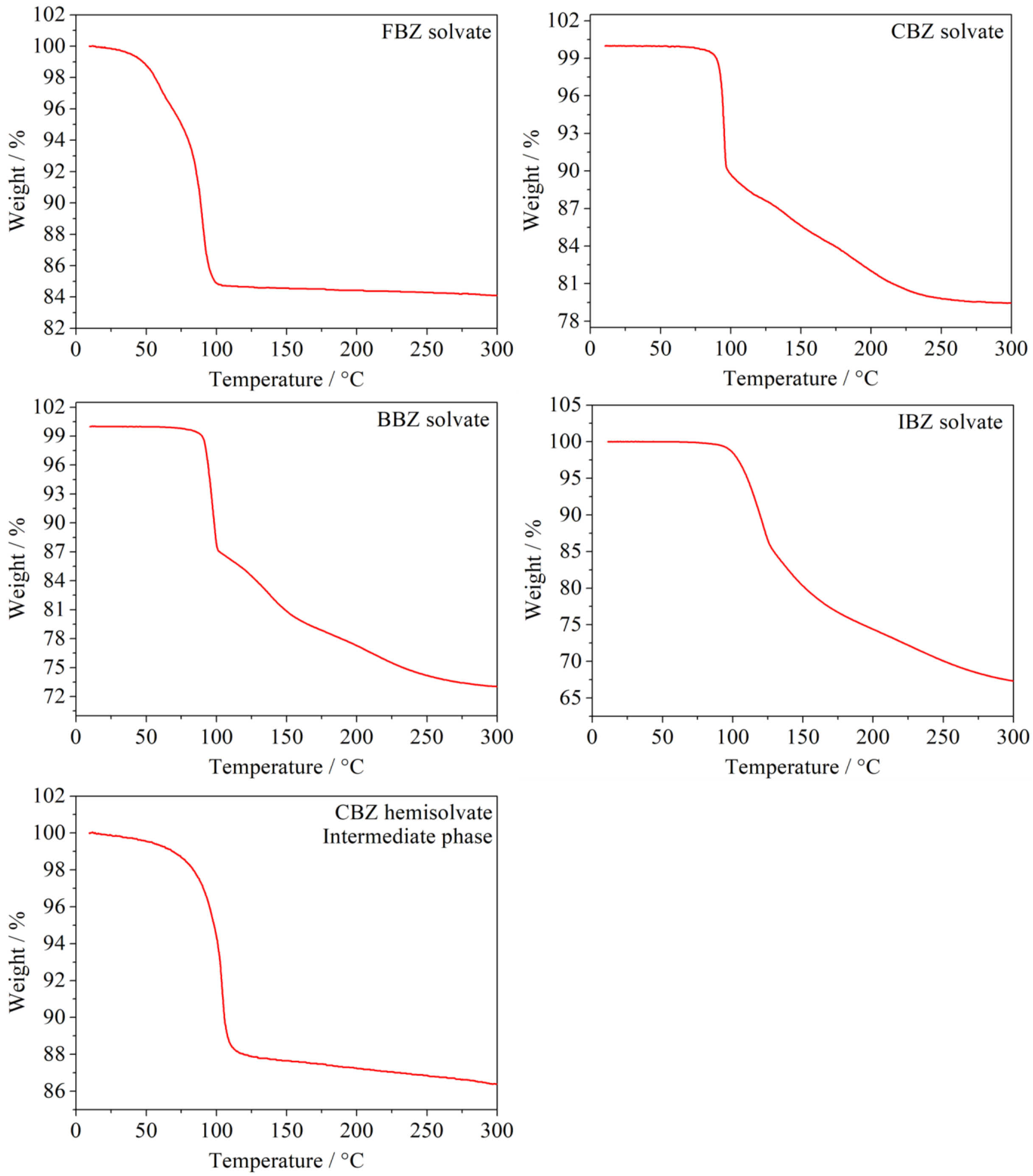

Figure S6. TGA curves of measured solvates.

Competitive slurry (nuclear magnetic resonance)

The ratio of solvents in each sample was evaluated using liquid nuclear magnetic resonance. The NMR spectra of fluorobenzene, bromobenzene, ibrutinib and sample J (table 1 in the manuscript) are portrayed in Figure S7 below. These spectra were used to evaluate the ratio of solvents in sample J (combination of FBZ and BBZ solvents) and are given as an example for all other samples, which were evaluated similarly.

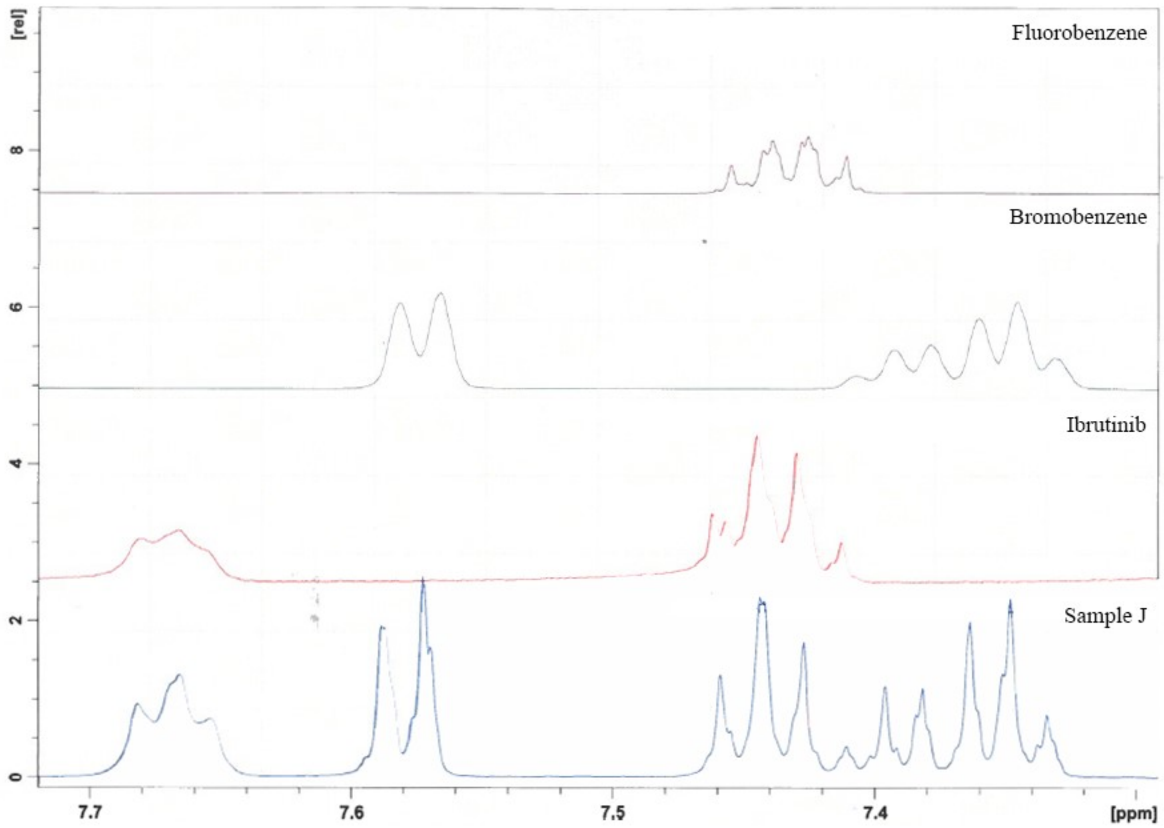

Figure S7. NMR spectra of fluorobenzene, bromobenzene, ibrutinib and sample J.

Ibrutinib amorphization in the presence of iodobenzene

The amorphization of crystalline ibrutinib form C in the presence of IBZ was described in the manuscript. It was shown using XRPD data. The phenomenon is further suggested by the behavior of the crystalline sample while in contact with the IBZ solvent. The sample forms small (approx. 0.5 mm in diameter), almost perfectly round spheres (Figure S8), which suggests amorphization of the sample. This is not a common behavior for crystalline materials.

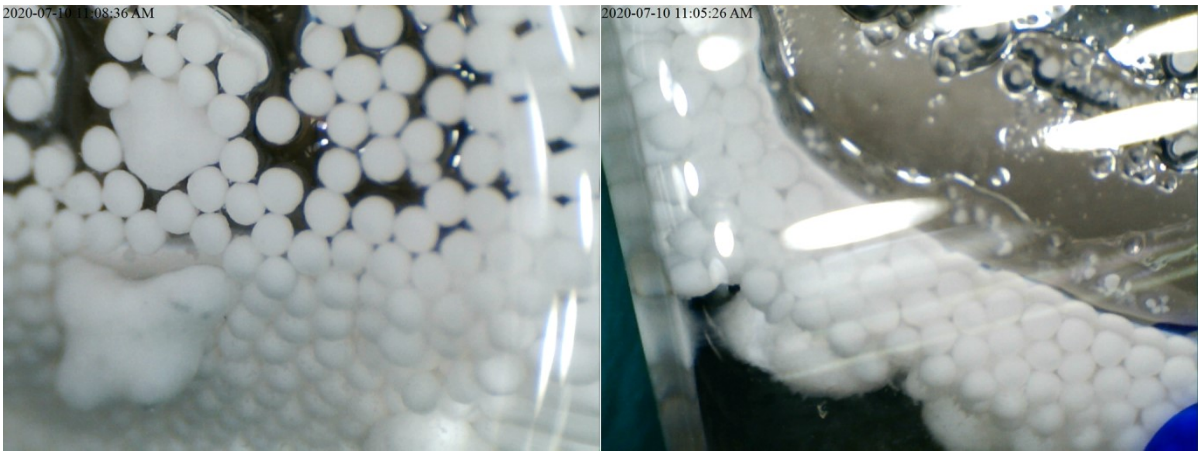

Figure S8. Small spheres formed after contact of ibrutinib form C with IBZ solvent.

Preparation of chlorobenzene hemisolvate (intermediate crystalline phase)

Ibrutinib (100 mg) was mixed with chlorobenzene (23 µl) and heptane (112 µl) in a vial. Ibrutinib and chlorobenzene are in 1:1 molar ratio. Heptane increases the overall volume of the solution and works as an inert liquid. Heptane homogenously disperses the molecules of chlorobenzene and slows down the reaction between ibrutinib and chlorobenzene. Ibrutinib C started to gradually transform to chlorobenzene hemisolvate. We have monitored the transformation using XRPD and once the diffraction pattern of ibrutinib C was no longer detectable, the experiment was terminated, solution filtered and powder dried. This powder was further used for crystal structure solution and further analysis.

Interaction energy calculations

The X-ray structures of all solvates (FBZ, CBZ, BBZ, and IBZ solvates; described above) and the ibrutinib itself (IBR; CCDC code 1559242) were optimized as fully periodic systems in the CASTEP program(Clark *et al.*, 2005) that uses pseudopotentials to model the effects of core electrons and plane waves to describe the valence electrons. We performed the optimization at the DFT level using the PBE(Perdew *et al.*, 1996) functional with the semi-empirical dispersion correction (G06 correction scheme by Grimme(Grimme, 2006)) and ultrasoft pseudopotentials. The plane-wave basis-set energy cutoff was set to 630 eV and the ultrafine SCF tolerance was used. The fine k-point set-up (3×2×2) was employed to sample the Brillouin zone via a Monkhorst–Pack grid(Monkhorst & Pack, 1976). Lattice parameters and its symmetry were fixed to the experimental values, while atomic positions were optimized. Additionally, we constructed unit-cells of a solvent containing two molecules of a solvent. These unit-cells were optimized using the same parameters as the solvates. We started from the solvate optimized structure and then deleted both molecules of ibrutinib. The atomic positions of the solvent molecules were then optimized while preserving the parameters of the unit-cell. Final enthalpies of all optimized structures were then used to obtain interacting energies of a solvate: $E_{int} = E_{solvate} - (E_{ibrutinib} + E_{solvent})$. A schematic representation of the approach is depicted in Figure S9. Alternatively, the solvent unit-cell was built with only one molecule of the solvent, and E_{int} was calculated as $E_{int} = E_{solvate} - (E_{ibrutinib} + 2E_{solvent})$. A similar approach was also adopted for the calculation of E_{int} of the CBZ intermediate. Such intermediate contains two molecules of ibrutinib and only one molecule of chlorobenzene in the unit cell and $E_{int} = E_{intermediate} - (E_{ibrutinib} + E_{CBZ})$, where E_{CBZ} corresponds to simulated CBZ unit-cell with one molecule of CBZ and the symmetry and dimension of the final CBZ solvate.

We also estimated E_{int} using a different approach based on isolated molecules and unit-cells using the Gaussian16 software(Frisch *et al.*, 2016). We calculated the single-point energy of the optimized unit-cells of all solvates and ibrutinib, as well as isolated molecules of the solvent, at the wB97XD level. The LANL2DZ effective core potential was used to describe F, Cl, Br, or I atoms, while the 6-311++G** basis set was used for the rest. Then the E_{int} was obtained as: $E_{int} = E_{solvate} -$

$(E_{ibrutinib} + 2E_{solvent})$ because the solvate unit-cells contain two molecules of ibrutinib and two molecules of the solvent. Analogously, the ibrutinib unit-cell contains two molecules of ibrutinib. Finally, we systematically constructed structures containing two optimized unit-cells in different *a*, *b*, and *c* directions (i.e. 2×1×1, 1×2×1, 1×1×2). Energies of these structures were then calculated at the PBE/3-21G and PBE/6-31+G**/LANL2DZ levels. Obtained energies were used to estimate the crystal elongation energies in each direction according to the formula: $E_{elongation} = E_{2\times1\times1} - 2E_{1\times1\times1}$, where $E_{1\times1\times1}$ is the energy of a single unit-cell. See the Figure S10 for better imagination.

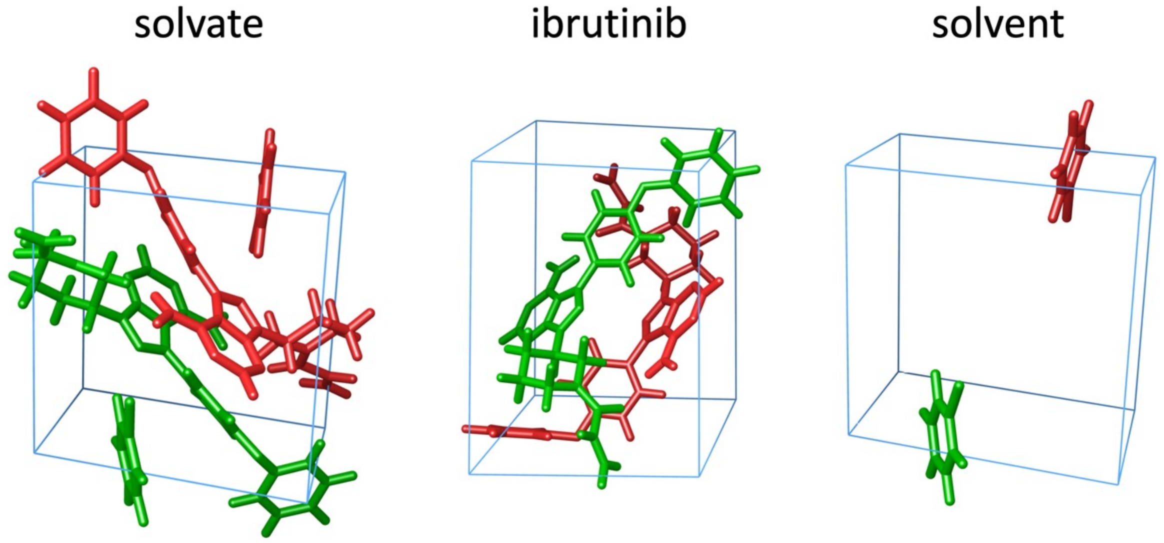

Figure S9. Schematic representation of crystal unit-cells considered in the calculation of E_{int} using CASTEP. Optimized unit-cell of the IBR-BBZ solvate is on the left, pure IBR in the middle and optimized BBZ on the right.

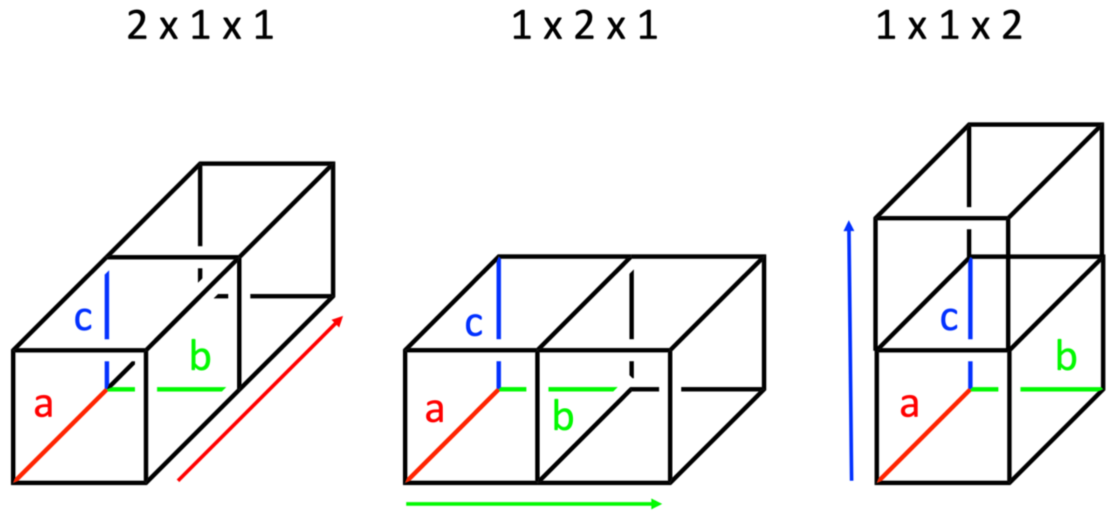

Figure S10. Schematic representation of the elongation assessment. The unit-cell was propagated in the *a*, *b*, and *c* direction, respectively, by an extra unit cell. The elongation energy was estimated as described above.

CrystalCMP

For the packing similarity comparison of the structures in the manuscript was used CrystalCMP. Since many readers might not be familiar with the software used for this purpose, there is a short description of the software. The CrystalCMP software works differently compared to the Crystal Packing Similarity tool used in Mercury (one of the tools many readers might be familiar with). The comparison of the clusters within Mercury is based on the calculations of the differences between the interatomic positions and the similarity/difference is calculated as positional difference between molecules in a molecular cluster. CrystalCMP on the other hand, calculates the packing similarity differently. The similarity is calculated from the differences between positions and rotations between the center of the related molecules in the cluster. The deviations of the positions and rotations are considered in the packing similarity (PS) as follows:

$$PS = D + X \cdot \frac{A}{180},$$

where D is the average distance (in Å) between molecular centers of related molecules and A is the is the average angle between them. X is a default parameter that weighs the influence of the A parameter (is set to 100 by default). The values of PS are plotted on the x-axis (fig. 5b in the manuscript) for the different solid forms showing the differences in the packing. The compared structures are more similar in terms of crystal packing the smaller the difference in PS is. For the calculations are used only the biggest molecule of the cluster (apremilast in this case), thus ignoring the guest molecule. For more details refer to the original publication(Rohlicek *et al.*, 2016).

Competitive slurry

Table S6. Summary of all presented solvent combinations with the notation used in the manuscript.

	Solvent			
Ibrutinib C	FBZ	CBZ	BBZ	IBZ
	4 solvent combination			
A	Yes	Yes	Yes	Yes
	3 solvent combinations			
B	No	Yes	Yes	Yes
C	Yes	No	Yes	Yes
D	Yes	Yes	No	Yes
E	Yes	Yes	Yes	No
	2 solvent combinations			
F	No	No	Yes	Yes
G	No	Yes	No	Yes
H	Yes	No	No	Yes

I	No	Yes	Yes	No
J	Yes	No	Yes	No
K	Yes	Yes	No	No

Aakeröy, C. B. & Salmon, D. J. (2005). *CrystEngComm* **7**, 439-448.

Aguiar, A. J. & Zelmer, J. E. (1969). *J. Pharm. Sci.* **58**, 983-987.

Amrutha, S., Giri, L., SeethaLekshmi, S. & Varughese, S. (2020). *Cryst. Growth Des.* **20**, 5086-5096.

Arlin, J.-B., Florence, A. J., Johnston, A., Kennedy, A. R., Miller, G. J. & Patterson, K. (2011). *Cryst. Growth Des.* **11**, 1318-1327.

Betteridge, P. W., Carruthers, J. R., Cooper, R. I., Prout, K. & Watkin, D. J. (2003). *J. Appl. Crystallogr.* **36**, 1487.

Billot, P., Hosek, P. & Perrin, M.-A. (2013). *Org. Process Res. Dev* **17**, 505-511.

Bolton, O., Simke, L. R., Pagoria, P. F. & Matzger, A. J. (2012). *Cryst. Growth Des.* **12**, 4311-4314.

Clark, S. J., Segall, M. D., Pickard, C. J., Hasnip, P. J., Probert, M. I. J., Refson, K. & Payne, M. C. (2005). *Z. Kristallogr. Cryst. Mater.* **220**, 567-570.

Collier, E. A., Davey, R. J., Black, S. N. & Roberts, R. J. (2006). *Acta Crystallogr., Sect. B: Struct. Sci., Cryst. Eng. Mater.* **62**, 498-505.

Dash, S. G. & Thakur, T. S. (2021). *Cryst. Growth Des.* **21**, 449-461.

Desiraju, G. R. (1995). *Angew. Chem. Int. Ed.* **34**, 2311-2327.

FDA Drug Approval Package: Imbruvica (ibrutinib), https://www.accessdata.fda.gov/drugsatfda_docs/nda/2018/210563Orig1s000,210563Orig2s000Approv.pdf.

Frisch, M. J., Trucks, G. W., Schlegel, H. B., Scuseria, G. E., Robb, M. A., Cheeseman, J. R., Scalmani, G., Barone, V., Petersson, G. A., Nakatsuji, H., Li, X., Caricato, M., Marenich, A. V., Bloino, J., Janesko, B. G., Gomperts, R., Mennucci, B., Hratchian, H. P., Ortiz, J. V., Izmaylov, A. F., Sonnenberg, J. L., Williams, Ding, F., Lipparini, F., Egidi, F., Goings, J., Peng, B., Petrone, A., Henderson, T., Ranasinghe, D., Zakrzewski, V. G., Gao, J., Rega, N., Zheng, G., Liang, W., Hada, M., Ehara, M., Toyota, K., Fukuda, R., Hasegawa, J., Ishida, M., Nakajima, T., Honda, Y., Kitao, O., Nakai, H., Vreven, T., Throssell, K., Montgomery Jr., J. A., Peralta, J. E., Ogliaro, F., Bearpark, M. J., Heyd, J. J., Brothers, E. N., Kudin, K. N., Staroverov, V. N., Keith, T. A., Kobayashi, R., Normand, J., Raghavachari, K., Rendell, A. P., Burant, J. C., Iyengar, S. S., Tomasi, J., Cossi, M., Millam, J. M.,

Klene, M., Adamo, C., Cammi, R., Ochterski, J. W., Martin, R. L., Morokuma, K., Farkas, O., Foresman, J. B. & Fox, D. J. (2016). *Gaussian 16 Rev. C.01.*

Grimme, S. (2006). *J. Comput. Chem.* **27**, 1787-1799.

Issa, N., Karamertzanis, P. G., Welch, G. W. A. & Price, S. L. (2009). *Cryst. Growth Des.* **9**, 442-453.

Karamertzanis, P. G., Kazantsev, A. V., Issa, N., Welch, G. W. A., Adjiman, C. S., Pantelides, C. C. & Price, S. L. (2009). *J. Chem. Theory Comput.* **5**, 1432-1448.

Kokubo, H., Morimoto, K., Ishida, T., Inoue, M. & Morisaka, K. (1987). *Int. J. Pharm.* **35**, 181-183.

Macrae, C. F., Sovago, I., Cottrell, S. J., Galek, P. T. A., McCabe, P., Pidcock, E., Platings, M., Shields, G. P., Stevens, J. S., Towler, M. & Wood, P. A. (2020). *J. Appl. Crystallogr.* **53**, 226-235.

Matsuda, H., Osaki, K. & Nitta, I. (1958). *Bull. Chem. Soc. Jpn.* **31**, 611-620.

Matsuda, Y., Akazawa, R., Teraoka, R. & Otsuka, M. (1994). *J. Pharm. Pharmacol.* **46**, 162-167.

Monkhorst, H. J. & Pack, J. D. (1976). *Phys. Rev. B* **13**, 5188-5192.

Musumeci, D., Hunter, C. A., Prohens, R., Scuderi, S. & McCabe, J. F. (2011). *Chem. Sci. J.* **2**, 883-890.

Nauha, E. & Nissinen, M. (2011). *J. Mol. Struct.* **1006**, 566-569.

Norbert Purro, M. S., Erick Goldman, David D. Wirth, (**2013**).

Palatinus, L. & Chapuis, G. (2007). *J. Appl. Crystallogr.* **40**, 786-790.

Pandit, J. K., Gupta, S. K., Gode, K. D. & Mishra, B. (1984). *Int. J. Pharm.* **21**, 129-132.

Perdew, J. P., Burke, K. & Ernzerhof, M. (1996). *Phys. Rev. Lett.* **77**, 3865-3868.

Price, S. L. (2014). *Chem. Soc. Rev.* **43**, 2098-2111.

Puig de la Bellacasa, R., Roué, G., Balsas, P., Pérez-Galán, P., Teixidó, J., Colomer, D. & Borrell, J. I. (2014). *Eur. J. Med. Chem.* **86**, 664-675.

Reilly, A. M. & Cooper, R. I. (2016). *Acta. Crystallogr. B. Struct. Sci. Cryst. Eng. Mater.* **72**, 439-459.

Rohlicek, J. & Husak, M. (2007). *J. Appl. Crystallogr.* **40**, 600-601.

Rohlicek, J., Skorepova, E., Babor, M. & Cejka, J. (2016). *J. Appl. Crystallogr.* **49**, 2172-2183.

Rohlíček, J., Zvoníček, V., Skořepová, E. & Šoóš, M. (2020). *Powder Diffr.* **35**, 160-165.

Rozovski, U., Hazan-Halevy, I., Keating, M. J. & Estrov, Z. (2014). *Cancer Lett.* **352**, 4-14.

S. de Moraes, L., Edwards, D., Florence, A. J., Johnston, A., Johnston, B. F., Morrison, C. A. & Kennedy, A. R. (2017). *Cryst. Growth Des.* **17**, 3277-3286.

Schultheiss, N. & Newman, A. (2009). *Cryst. Growth Des.* **9**, 2950-2967.

Sládková, V., Skalická, T., Skořepová, E., Čejka, J., Eigner, V. & Kratochvíl, B. (2015). *CrystEngComm* **17**, 4712-4721.

Stanton, M. K. & Bak, A. (2008). *Cryst. Growth Des.* **8**, 3856-3862.

Sun, C. & Grant, D. J. W. (2001). *Pharm. Res.* **18**, 274-280.

Suresh, K., Minkov, V. S., Namila, K. K., Derevyannikova, E., Losev, E., Nangia, A. & Boldyreva, E. V. (2015). *Cryst. Growth Des.* **15**, 3498-3510.

Vasilopoulos, Y., Heyda, J., Rohlíček, J., Skořepová, E., Zvoníček, V. & Šoóš, M. (2022). *J. Phys. Chem. B* **126**, 503-512.

Veeraraghavan, S., Viswanadha, S., Thappali, S., Govindarajulu, B., Vakkalanka, S. & Rangasamy, M. (2015). *J. Pharm. Biomed. Anal.* **107**, 151-158.

Wöhler, F. (1844). *Annalen Chem. Pharm.* **51**, 145-163.

Young, R. M. & Staudt, L. M. (2014). *Cancer Cell* **26**, 11-13.

Zvoníček, V., Skořepová, E., Dušek, M., Babor, M., Žvátora, P. & Šoóš, M. (2017). *Cryst. Growth Des.* **17**, 3116-3127.

Zvoníček, V., Skořepová, E., Dušek, M., Žvátora, P. & Šoóš, M. (2018). *Cryst. Growth Des.* **18**, 1315-1326.
